# Establishment of a reborn MMV-microarray technology: realization of microbiome analysis and other hitherto inaccessible technologies

**DOI:** 10.1186/1472-6750-14-78

**Published:** 2014-08-21

**Authors:** Harshita Sharma, Yasunori Kinoshita, Seiichi Fujiu, Shota Nomura, Mizuho Sawada, Shamim Ahmed, Masaki Shibuya, Kosaku Shirai, Syota Takamatsu, Tsuyoshi Watanabe, Hitoshi Yamazaki, Ryohei Kamiyama, Tetsuya Kobayashi, Hidenao Arai, Miho Suzuki, Naoto Nemoto, Ki Ando, Hidekazu Uchida, Koichiro Kitamura, Osamu Takei, Koichi Nishigaki

**Affiliations:** 1 Department of Functional Materials Science, Graduate School of Science and Engineering, Saitama University, 255 Shimo-okubo, Saitama 338-8570, Japan; 2 Janusys Corporation, Saitama Industrial Technology Center, 3-12-18 Kamiaoki, Kawaguchi, Saitama 334-0844, Japan; 3 Lifetech Co., Ltd., 4074 Miyadera, Iruma City, Saitama 358-0014, Japan; 4 Enplas Corporation, 2-30-1 Namiki, Kawaguchi City, Saitama 332-0034, Japan; 5 Finetech Corporation, 1-7-1, Asagaya-minami Suginami-ku, Tokyo, Japan; 6 Present address: Department of Biochemistry and Molecular Biology, Shahjalal University of Science and Technology, Sylhet, Bangladesh; 7 Present address: Department of Electrical and Electronic Engineering, Tokyo Denki University, 5 Senjyu-Asahi-cho, Adachi-ku, Tokyo 120-8551, Japan

**Keywords:** Microarray, Multi-parallel reactions, Multi-conditioner, Lysozyme crystallization, Microbiome analysis

## Abstract

**Background:**

With the accelerating development of bioscience, the problem of research cost has become important. We previously devised and developed a novel concept microarray with manageable volumes (MMV) using a soft gel. It demonstrated the great potential of the MMV technology with the examples of 1024-parallel-cell culture and PCR experiments. However, its full potential failed to be expressed, owing to the nature of the material used for the MMV chip.

**Results:**

In the present study, by developing plastic-based MMVs and associated technologies, we introduced novel technologies such as C2D2P (in which the cells in each well are converted from DNA to protein in 1024-parallel), NGS-non-dependent microbiome analysis, and other powerful applications.

**Conclusions:**

The reborn MMV-microarray technology has proven to be highly efficient and cost-effective (with approximately 100-fold cost reduction) and enables us to realize hitherto unattainable technologies.

## Background

For drug discovery, high-throughput screening is ultimately required. This is similar to those in various other scientific fields in which optimal conditions and combinations must be verified. The rising cost of reagents has motivated the development of microminiaturization technologies such as microarrays and microfluidics. For example, microplates have evolved from 96–384 wells to up to 3456 wells, lowering assay cost owing to the reduction of the amount of reagents and samples [1,2]. However, even the 3456-well plate requires μL volumes for handling, due to adsorption and evaporation losses.

In recent years, many digital PCR systems such as the Fluidigm and OpenArray systems have provided a leap toward the miniaturization of assay vessels by permitting gene expression analysis at the nL scale [3,4]. Moreover, picoliter-droplet digital PCR system is capable of analyzing millions of picoliter-sized droplets for molecular genotyping [5]. However, these systems are highly specialized for specific purposes, requiring specific liquid handlers and digital PCR instruments [6]. In addition, multistep reactions are, in principle, difficult to perform on these platforms, confining their applicability to molecular genotyping and a few other assays.

Microfluidics and bead-based technologies have been developed and are currently still expanding their possibilities [7-10]. These technologies have the potential to deal with minute amounts of sample and, particularly in microfluidics, support multistep reactions and detection. However, there are some difficulties in microfluidics in supporting the high density of parallel operations owing to the difficulty in supplying a uniform force to drive liquids [11], avoiding losses from surface adsorption, and other operations [12,13]. In bead technology, parallel bead handling requires highly specialized machines that cannot be readily used for general purposes [14], although they are useful for specific purposes such as random multiple sequencing [15]. In summary, current innovative methods have their own merits but require further development to reach their goal such as high cost performance.

Here, we describe a reborn MMV-microarray technology, i.e., microarray with manageable volumes, operated without pipettes. Pipette-free operation offers several benefits, for instance, rapid operation, handling of nanoliter volumes, avoiding adsorption and evaporation losses during operations, and dispensing with elaborate equipment. This technology was originally developed using polyacrylamide gel [16,17] and had the main drawbacks of fragility and difficulty of accurate handling, although it has already enabled us to culture bacteria and perform PCR reactions [17]. The method reported here could overcome these defects, thereby strengthening the MMV technology.

Microarray technology has permitted a high density of parallelism and the development of various methods [18,19]. However, this technology has the constraint that it essentially comprises surface reactions and is difficult to use for successive and independent multistep reactions. The MMV technology solves this problem and has a potential to support novel applications. In the present study, this potential to enhance the basic MMV technology is described.

## Results

### Sub-μL liquid handling technique

**Figure 1:**
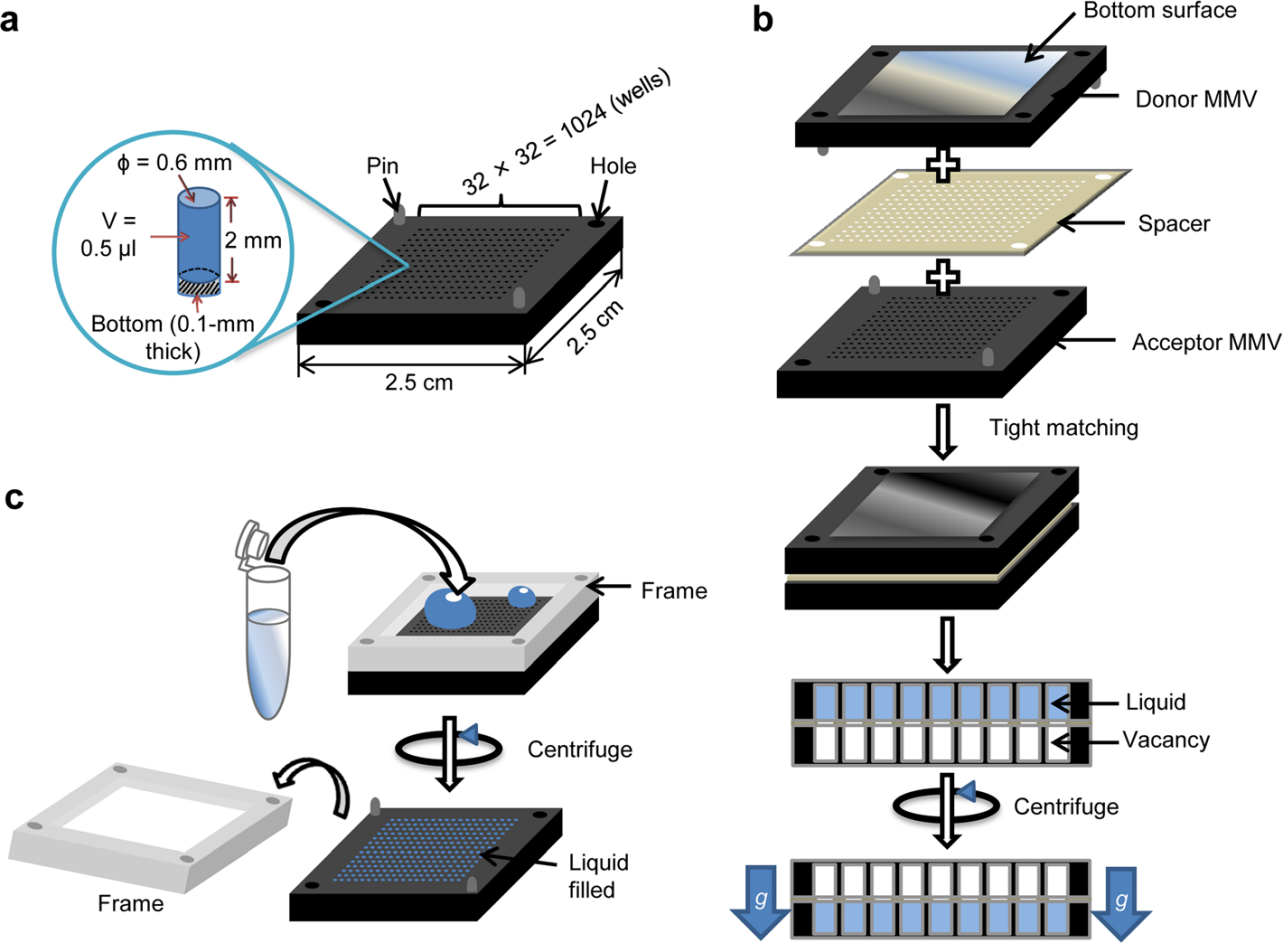
**MMV chip construction and basic operation. (a)** Dimensions of an MMV chip. The MMV chip has a dimension of 2.5 cm × 2.5 cm × 0.2 cm and is fabricated with 1024 wells of 0.6-mm diameter and 2-mm depth, giving a volume of 0.5 μL of solution. **(b)** Usual transfer (Z-mode) of solution. Well-to-well transfer of solution is performed using a silicone/urethane spacer that enables a leak-proof flow of solution from the donor to the acceptor MMV. **(c)** Schematic representation of initial input of solution into wells (I-mode). A urethane/silicone frame is used to confine the solution to the top surface of the MMV.

In this study, we developed novel necessary tools and principal technologies for pipette-free sub-μL volume handling in MMV, which were not available for MMV made from gel (Figure 1a). The main principle of MMV is “aperture-to-aperture transfer.” Parallel transfer of thousands of solutions was performed by centrifugation of a face-to-face stack of two MMVs with each well tightly contacting the other via an adhesive spacer (Figure 1b). Initial charging with sample was performed as shown in Figure 1c. Similarly, various operation techniques have been developed, including those described in our previous report [17], and are collectively presented in Table 1 (for detail, see Additional file 1: Table S1, Additional file 2: Figure S1, Additional file 3: Figure S2, Additional file 4: Figure S3, Additional file 5: Figure S4, Additional file 6: Figure S5, Additional file 7: Figure S6, Additional file 8: Figure S7, Additional file 9: Figure S8, Additional file 10: Figure S9).

**Table 1:** Basic transfer operations needed for MMV

**Operation (Pipette-dependence mode**^**a**^**)**				**Purpose**	**Details**
**PF**	1	Input (I-mode)		Liquid charging into MMV wells	Figure 1c
	2	Output (O-mode)	a. Transfer	Complete ejection of liquid in wells	Figure 1b and Additional file 6: Figure S5
			b. Washing		
	3	Square transfer (S-mode)		Fractional volume transfer	Additional file 2: Figure S1
	4	Z-direction transfer (Z-mode)		Normal transfer of liquid into wells	Figure 1b and Additional file 5: Figure S4
	5	X-direction transfer (X-mode)		Division of liquid in all wells	Additional file 7: Figure S6
	6	Magnetic beads transfer (M-mode)	a. Beads recovery	Beads recovery	Additional file 8: Figure S7
			b. Division of liquid	Division of liquid in all wells	Additional file 9: Figure S8
	7	Filtering (F-mode)		Filter-selective transfer	Figure 2a
**PD**	8	Pipette-dependent transfer (P-mode)	a. Manual	Manual transfer of liquid or beads	Additional file 10: Figure S9
			b. Robotic	Robotic transfer of liquid or beads	Additional file 3: Figure S2

^**a**^Pipette-free (PF) or pipette-dependent (PD).

**Figure 2:**
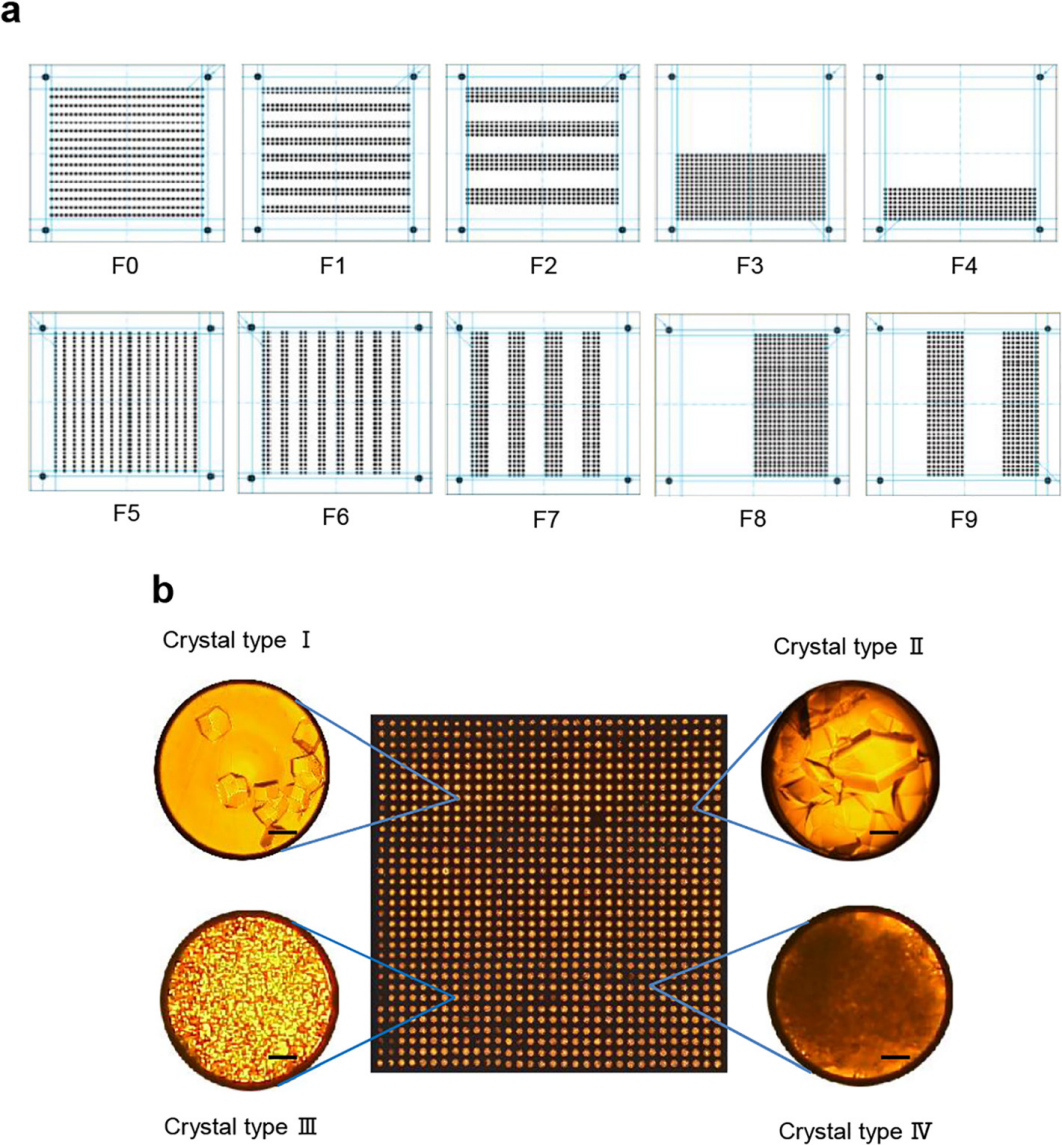
**Generation of 1024 different conditions. (a)** A set of filters used to generate multiple conditions in an MMV chip. In each of these filters, if the N-bit is 1 (“go”) for the (*i* + 1)-th least significant bit in the binary number, then the corresponding position of filter F_i_ is a hole (allowing the liquid to pass through). On selective addition of 10 species of materials to an acceptor MMV using these filters, 1024 different conditions (corresponding to 1024 different binary numbers) were generated (for detail, see Additional file 11: Figure S10). **(b)** Lysozyme crystallization in MMV. The 1024 different conditions were generated for determining the optimum crystallization conditions. A combination of pH differences (2.9–9.6) and NaCl concentration differences (0–1.5 M) was examined (see Methods). In the inset, magnified images are shown for some MMV wells. The scale bar is 100 μm.

For example, selective transfer of solutions (F-mode) to specific wells was achieved by exploiting specifically patterned filters (Figure 2a). In the MMV square transfer (S-mode) method, different volumes of solutions were transferred using the MMV-matching thin filter device (Additional file 2: Figure S1). Although in general, “well-to-well transfer” had been adopted in the MMV technology and could accomplish most necessary operations, we also developed an automated liquid handling (P-mode) system to achieve versatility and manageability, expanding the ability of the MMV system (Additional file 3: Figure S2).

### Generation of replicas of cell-to-cell (C2C) and DNA-to-DNA (D2D)

**Figure 3:**
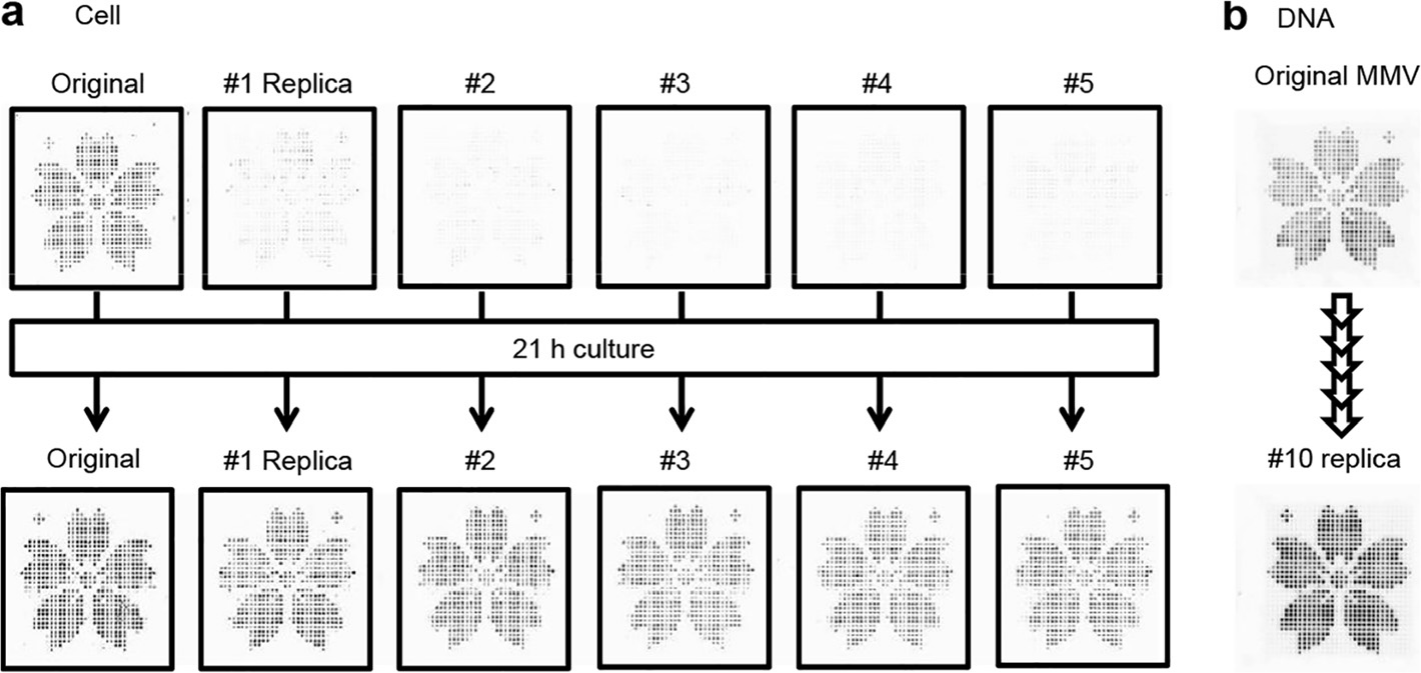
**Generation of the MMV replica. (a)** Cell replica. The liquid in the original MMV containing *E. coli* cells (harboring the GFP plasmid) in which the wells collectively resemble a cherry blossom pattern was transferred and returned, leaving a minute droplet in each replica MMV (upper rows #1 ~ 5). All these MMVs were added with culture media and incubated at 37°C for 21 h to regenerate cells (lower row). **(b)** DNA replica. The replica image is that of the 10th replication (PCR amplified), whereas the original MMV is shown just after the 10 rounds of replica generation operation. Both are stained with SYBR green I.

To demonstrate the efficient multistep parallel transfer of solutions, MMVs containing DNA and cells in desired patterns were fabricated and used to test replica generation (Additional file 4: Figure S3a and b). The MMV chip containing *E. coli* cells in a pattern (cherry blossom) was subjected to successive five rounds of forward [from the donor MMV (original) to the acceptor MMV (replica)] and backward transfer [transfer back of solution from the acceptor MMV (replica) to the donor MMV (original)] with replacement of each acceptor MMV chip. The vacant acceptor chips were then filled with Davis media and incubated at 37°C for 21 h. As shown in Figure 3a, every replica chip reproduces the original cherry blossom pattern beginning with the vacant state, demonstrating the effectiveness of the replica generation technique. As the original itself can be readily amplified, we can produce replicas perpetually. Close observation of these patterns indicated that 1% or fewer of the wells stochastically failed to amplify cells within this incubation time. In an auxiliary experiment, a very small amount of aliquot (approximately 0.1% of the total solution) was shown to be left in each vacant well (Additional file 4: Figure S3c and d) and served as a seed for the next bacterial culture. Therefore, although the current system is subject to stochastic errors at a level of 0.1%, it can be used with careful monitoring of each well's state (presence of an aliquot) under a microscope. Otherwise, it can be safely used by making multiple copies (3–4) of each sample. This situation can be greatly improved with the use of a much finer, though more expensive casting mold to generate a next-generation MMV chip.

Similar repetitive forward and backward transfer experiments were performed with DNA solution, and vacant MMV chips were filled with a reaction buffer and then subjected to PCR. As shown in Figure 3b, the 10th replica was completely replicated, showing that a DNA replica array was also successfully generated. In an independent experiment, cross-contamination between wells was shown to be eliminated by the introduction of a packing spacer (of the same shape as the MMV chip and with a thickness of 0.8 mm) (Figure 1b and see Additional file 5: Figure S4 for actual unit transfer). To our knowledge, this is the first success in perpetual DNA microarray replica formation (DNA microarray replication effective only once for all was reported in a previous study) [20].

### Generation of 1024 conditions (multiple-conditioner)

To exploit the array nature of MMV, we generated 1024 different conditions by applying the “2^N^ method” [17] (Additional file 11: Figure S10 and Additional file 12). To investigate the crystallization conditions of lysozyme, ten types of template MMVs were used to generate 2^10^ (=1024) different conditions (Figure 2a). The conditions ranged from pH 2.9 to 9.6 and from 0 to 1.5 M NaCl (Additional file 13: Figure S11a). Typical crystal images that were obtained are shown in Figure 2b and Additional file 13: Figure S11, and they comprised type I (0.1–0.2-mm large tetragonal), type II (tetragonal larger than 0.2 mm), type III (0.03 mm or smaller microcrystalline), and type IV (needle-like) crystals. This result showed the most optimal conditions (pH 8.6 and 0.6 M NaCl) for generating the largest crystal (0.65 mm; Additional file 13: Figure S11a and b) of lysozyme as well as the conditions under which other types of crystals can be obtained. This crystal size of lysozyme seems to be relatively large, although one of 1.6 mm has recently been reported [21]. The sample amount required for a single condition was in the mg range for that experiment, but only 10 μg (100-fold smaller) for ours.

### Microbiome analysis without dependence on NGS

Identifying all the members of microbiome found in an ecosystem is important for genuine understanding. Accordingly, next-generation sequencing (NGS), which is a powerful, but expensive technique has been used for microbiome analysis [22-24]. The amount of information obtained using this method is often too great for the mere identification of organisms.

**Figure 4:**
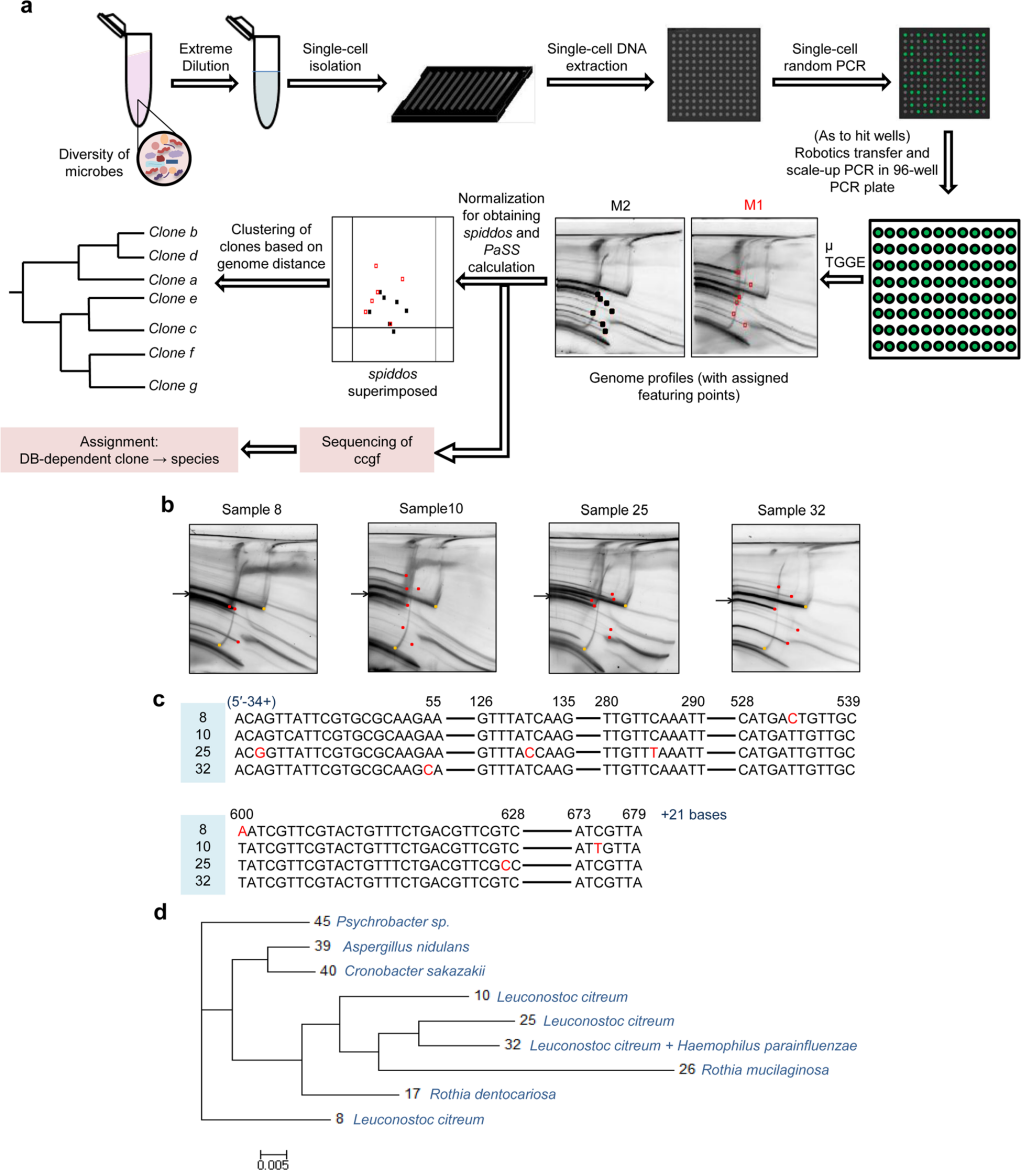
**Schematic representation of NGS-non-dependent microbiome analysis (NNMA). (a)** A sample containing a complex mixture of microbes was serially diluted to a concentration corresponding to one or fewer cell/well as expectation value (in Poisson distribution). DNA extraction and random PCR were performed on the same MMV chip. The PCR product was scaled up using PCR in a 96-well plate and analyzed using μTGGE. In the genome profile obtained, feature points were assigned and processed using computer-aided normalization, generating species identification dots (*spiddos*). Based on *spiddos*, a genome distance, *d*_*G*_ [defined as 1 − *PaSS* (pattern similarity score)]_,_ was obtained for each pair of microbes (Additional file 12), and a clustering tree was then generated using *d*_*G*_. **(b)** Genome profiles of four samples with feature points assigned. Red dots represent feature points (pre-*spiddos*) and yellow dots, internal reference points. Arrow shows possible commonly conserved genetic fragment (ccgf). **(c)** Partial sequences of ccgfs. Point mutations are shown with red letters. Completely matching regions are shown with lines. **(d)** Clustering tree for nine samples. Here, tentative microbial species are assigned from the sequence obtained for ccgf. The tree was constructed using Phylip 3.69 and MEGA 5.1 software.

Another method could identify the species of organisms with cost-effective performance, i.e., genome profiling (GP) [25]. In this study, we investigated the possibility of MMV-dependent GP analysis of microbiomes. Its entire procedure is shown in Figure 4a. Several crucial features are included in this method; they are as follows: *i*) limiting the dilution of organisms constituting a microbiome entity into a single cell, *ii*) performing single-cell PCR by random PCR, *iii*) performing GP analysis using micro-temperature gradient gel electrophoresis (μTGGE), and *iv*) obtaining the sequence information of each cloned organism. The first feature can be achieved with the aid of multiple MMV microvessels to reduce the quantity of reagents required. In this study, dispersion of some of the flocculated bacteria was found to be a problem. However, this problem was substantially addressed, as shown in Methods (enzymatic treatment and sonication, although approximately 1% of the number of flocs remained after this treatment). The second feature addresses the difficulty of single-cell PCR [26] and the lack of a common PCR primer for all microbes. Fortunately, random PCR is in effect, which is a universal PCR that can be applied to any organism using the same primer [27]. Thus, we could easily overcome this problem. Much experience with single-cell PCR has been accumulated both worldwide [28-30] and in our laboratory [26].

Thus, it is no longer difficult to perform single-cell PCR, if precautions are taken to avoid adsorption loss and DNA contamination. The third feature (GP analysis using μTGGE) is supported by prior experience [25,26,31-34]. The final feature can be realized by another benefit of the GP method, i.e., the existence of easily assignable (from its band pattern in the electrophoresis) and PCR-recoverable DNA, namely, a commonly conserved genetic fragment (ccgf) [31] that can be amplified from DNA extracted from a gel band using the same primer adopted for random PCR.With solutions to each technical challenge, oral microbes were used as a test case and subjected to the entire NGS-non-dependent microbiome analysis (NNMA) shown in Figure 4a.

Figure 4b clearly demonstrates that some MMV wells generated distinct genome profiles (original data in Additional file 14: Figure S12) by the GP method, suggesting a successful microbial cell distribution in different MMV wells and extraction and amplification of DNA in each well. The DNAs recovered from the μTGGE gel as ccgfs were further analyzed by conventional cloning and sequencing analysis, and the resulting DNA sequences were subjected to BLAST analysis (Figure 4c). In this analysis, *Leuconostoc citreum*, *Haemophilus parainfluenzae*, *Rothia dentocariosa*, *R. mucilaginosa*, *Aspergillus nidulans*, *Cronobacter sakazakii*, and *Psychrobacter sp.* were tentatively identified based on sequence similarity with organisms in the NCBI database (see Additional file 15: Table S2). Because all these microbes can be assigned as stable or transient oral inhabitants, this finding strongly supported the inference that NNMA analysis was performed without obstacles.

The genome profiles and species identification dots (*spiddos)* that were obtained in this analysis (Figure 4b) were subjected to clustering analysis (Figure 4d), in which clones were assigned to possible species based on their own ccgf DNA sequence (Figure 4c and Additional file 15: Table S2).

In this experiment, not all but only some clones (47 of them) appearing in an MMV plate were processed to test the feasibility of the entire NNMA process because this number was sufficient to confirm experimental success. The same microbiome analysis was performed three times, with all tests performed using possible clones and their genome profiles (*spiddos*) (Additional file 16: Table S3), thereby supporting the effectiveness of the NNMA method.

## Discussion

In this paper, we have established the basic technology for the MMV handling and three typical applications of the MMV method: replica generation, generation of 1024 conditions, and microbiome analysis. The original solution (containing DNA or cells) can be repeatedly used for replica generation. According to the requirement, replicas containing defective wells can be repaired by the operation of a nanoliter-dispensing robot (see Additional file 3: Figures S2 and Additional file 17: Figure S13). In effect, this technology enables us to produce DNA, cell, and even protein arrays easily and endlessly. The quality of the replica thus generated is sufficiently reliable if MMV is loaded with samples in multiples of 10 (because the expected defect rate is <1 in 10, implying that more than 9 of 10 wells are intact). In addition, the use of a higher-quality MMV chip mold (a matter of cost) is empirically known to improve the defect rate greatly. Moreover, monitoring the content of each individual well (parameters such as volume, temperature, and conductivity) is within our scope and achieved by semiconductor technology.

Another important point is that the success of cell-to-cell (C2C) and DNA-to-DNA (D2D) replications implies that by combining cell-to-DNA (C2D) (Figure 4) and DNA-to-peptide/protein (D2P) [17] (Additional file 18: Figure S14), we can achieve C2D2P, thus beginning with cells and ending with a protein (peptide) microarray, an outcome that was never attainable before.

We generated 1024 conditions for lysozyme crystallization. This application of MMV is unexpectedly powerful and widely applicable because it circumvents lengthy and time-consuming preparation processes and spares reagents. The crystallization study described here was performed only to demonstrate the utility and capacity of the 1024-well MMV technology (because such research has already been performed [17,21,35-37]), and to show that a high volume of information can be readily obtained with high performance. In this experiment, we obtained crystals that were larger than 200 μm in a much smaller volume (0.5 μL) than that used in previous studies [21,35,36], thus sparing samples and reagents by about 100-fold in comparison with conventional approaches, although a more competitive (∼150-fold) reduction technique has been reported that employs 81 microwells and a specially fabricated apparatus [37]. Additionally, we evaluated the reproducibility of the optimal crystallization conditions at the 50 μL scale, resulting in crystals that were around 0.65 mm long (Additional file 13: Figure S11b). If necessary, a more statistically reliable and realistic approach can be applied to examine each condition in quadruplex or octuplex. Even in an octuplex test, more than 100 conditions can be readily examined in a single MMV chip test, with only 4 μL (0.5 μL × 8) consumed per condition.

The conditions that can be examined are not only those for crystallization of proteins, but also those for the combinatorial effect of biological factors (hormones, cytokines, and others) on induction of cell differentiation such as iPS (induced pluripotent stem cell) induction. In the field of medicinal chemistry, more effective drug combinations (synergy effect) may be easily found owing to the readiness in generating multiple conditions with ease and low cost. Because chemical and biological experiments originally require surveys over a wide experimental range and multiple assays, the problems of cost, labor, and time-consuming processes, which have inhibited desirable experiments, can be revolutionarily solved by the MMV approach.

Furthermore, the NNMA system is a potential method for identifying the component species of microbiomes. To date, we have had no effective method to analyze a huge microbiome population mainly because we lacked an appropriate container to accommodate a large number of microbes separately and efficiently, i.e., accommodating a single organism per well and enabling a series of DNA extraction processes, PCR amplification, and DNA sequence analysis in multiples with an affordable cost in reasonable time. This situation was, for the first time, overcome by the advent of NGS, a technology worthy of the intensive attention of microbiologists. However, NGS is not always effective; it is very expensive, and the raw data generated is generally too difficult to process except by bioinformatics specialists. A significant amount of data accordingly remains unprocessed. In contrast, the NNMA system enables microbiologists to design and complete their study on their own.

The following potential advantage of NNMA over NGS further supports this approach: NNMA can deal with an organism as a whole, whereas NGS yields a mixture of genome fragments for analysis. Thus, the latter renders it impossible to further investigate the organism, as it is theoretically difficult to detect extreme minorities such as one or fewer in 1000, whereas NNMA offers the possibility of detecting, in principle, a minority of 1 in 10000 if twenty or more 1024-well MMV plates are used in the dilution with the expectation of 0.5 cell/well. This is the essential difference between the NGS and NNMA approaches.

A few points remain to be improved for the NNMA analysis, including an increase in the output of μTGGE with sample scale reduction. The realization of scale reduction will eliminate the scale-up step required in the current NNMA process (Figure 4a). Another goal is to develop a means of complete deflocculation (although this is a classical and difficult problem [38,39]), because in the present experiment, flocs were still present (Additional file 19: Figure S15), consistent with our observation that DNAs from a single well of MMV are sometimes assigned to different species (Additional file 15: Table S2). This observation is noteworthy because the current NNMA system, although it remains to be improved in techniques outside the MMV technology proper, has already succeeded in extracting DNAs from microbes, from either single or flocculated cell(s), and amplifying, analyzing, and assigning them to species using MMV-based technology—a feat never achieved by conventional approaches such as microplate-based technologies.

In this study, all necessary basic skills and tools for the MMV operations have been described. Moreover, highly usable application methods have been proposed.

In addition, there are other potentially powerful applications. Among them are the function-based selection of possible drug molecules termed as panning on microarray MMV (POMM) (Additional file 18: Figure S14) and All-In-One and All-At-Once (A^I^/_A_O) (Table 2). The former (POMM) is a multistep reaction experiment, comprising PCR, replica formation, enzymatic reactions (transcription/translation), and fluorescence detection supported by multiple operations of well-to-well transfers. The latter is simple and wide ranging, such that the diversities of its usage are open to researchers, particularly when pre-charged MMVs with various samples such as buffers, DNAs, enzymes, bead-bound antibodies, and cells are available from commercial sources in the future. If necessary, all one has to do is to purchase a pre-charged MMV with diverse conditions and overlay it on an experimenter's MMV (containing one's own samples). Similarly, an analyst can identify the optimal medium for culturing a particular cell (iPS or cancer cell) using an MMV of a combinatorial set of factors (generated by the 2^N^ method).

**Table 2:** MMV applications developed to date

**Application**	**Content**	**Level achieved**^**b**^	**Comment**
Multiple (1024) conditions generation (Multiple-conditioner)	Generation of 1024 different conditions in MMV for lysozyme crystallization.	+++	Applicable to iPS primary induction factor screening
Semi-infinite replica Formation (D2D, C2C, and D2D2P)	Replication of DNA and cells in MMV perpetually. Besides, DNA processed to protein (D2D2P) replica.	+++	Proteins replica can be perpetually generated by two steps of D2D and D2P, i.e., D2D2P.
NGS-non-dependent microbiome analysis (NNMA)	Single-cell isolation, DNA extraction, single-cell random PCR of microbiome samples in MMV, and processing of PCR products by the Genome Profiling (GP) method.	+++	Serves as single cell isolation and analysis tool
Multistep function-based screening (POMM)	Operation of all steps involved in DNA amplification, *in vitro* transcription and translation; identification of functional peptides in MMV.	++	Screening tool with samples addressed
All-In-One/All-At-Once assay (A^I^/_A_O)	Screening of apoptosis-inducing peptides against cancer cells.	+	Applicable to monoclonal antibody screening

^**b**^+++, successfully achieved; ++, whole process developed with tentative confirmation; +, system developed with successful preliminary experiment.

The MMV technology has the merits of simple operation, rapidity, economy, reduced error rate, and flexibility to both classical microscope-based technologies (phase-contrast, fluorescence, and others) and emerging semi-conductor-based technologies (Additional file 20: Figure S16).

## Conclusions

The plastic-based MMV chip enabled us to develop an easy-to-handle and quantitative system in handling sub-μL volumes. It also facilitated the development of a set of necessary unit technologies for MMV operation such as input, output, division, filtering of solutions, and others. This development has led to genuinely novel methods such as 1024-parallel Cell-to-DNA-to-Protein/Peptide (C2D2P) and NNMA.

In addition, MMV can reduce experimental time and cost drastically (Additional file 21: Table S4).

## Methods

The most basic nature of the MMV technology can be represented by the facts that the liquid in an MMV well does not fall by the gravity and is less volatile due to the narrow aperture area, easily thermo-conductive due to the small volume, and readily mixed by convection.

### MMV chip

Polycarbonate (PC) MMV chips were developed in a joint project, involving most of the authors, which was sponsored by JST, and were finally obtained from Enplas Corporation. The MMV chip has an overall dimension of 2.5 × 2.5 cm^2^, with diameter, depth, and volume of 0.6 mm, 2 mm, and 0.5 μL, respectively (Figure 1a). PC MMV is classified into the two following types according to the chip's bottom: *i*) PC molded and *ii*) transparent sheet pasted. For microscopic observation, the second type is used. Silicone rubber or PDMS was also used for fabricating flexible well-size MMV chips [17].

### Charging and discharging of liquid into MMV (I-mode)

Initial charging of sample into wells can be achieved by centrifugation force as shown in Figure 1c; with excess solution covering the top surface of MMV, it was centrifuged at 1400 *g* for 1 min to fill all the wells with solution and evacuate the remaining solution from MMV wells (Figure 1c).

As I-mode is a kind of dump transfer, a specific one-by-one transfer is carried out by P-mode as written in Table 1. In order to prevent evaporation, MMV was sealed with silicone tape (Rescue Tape), and to remove liquid from MMV wells, centrifugation was applied to the MMV placed upside down (at 1400 *g* for 2 min) (Additional file 6: Figure S5). One MMV chip can be repeatedly reused by several rounds of washing with ultrapure water and/or 70% ethanol by centrifugation and ultrasonic vibration. Liquid can be transferred from one well of an MMV to another well of an opposing MMV by centrifugation. A silicone-PET-adhesive spacer (0.2-mm thick with 0.6-mm wide holes) (Finetech) was fixed on the donor MMV and then the acceptor MMV was firmly laid against it (Figure 1b). The liquid moves from the donor to the acceptor MMV through the thin channel in the spacer without evaporation or adsorption losses.

Further, to add a droplet solution, S-mode operation (Additional file 2: Figure S1) was applied. This type of spacer was repeatedly used, and, if necessary, the thickness and/or hole patterns of the spacer were changed. Layers from bottom to top, namely MMV (used as support), a bottom-side-adhesive film attached to the MMV, and a spacer of desired thickness and hole pattern, were constructed and matched to charge the wells with the solution in the square method.

### Selective addition to specific wells (F-mode)

For this purpose, different types of filter spacers were produced (Finetech) and used (examples in Figure 2a). These spacers are adhesive and contain different surface patterns of holes and without holes, enabling selective transfer of samples to the acceptor MMV. As an alternative solution, template MMVs having a specific pattern of open and closed wells can be used for the same purpose. Such templates could be formed using PDMS and a laser patterning machine [17].

### MMV coating

To prevent adsorption of biomolecules on MMV surfaces, MMVs were mainly surface coated with a solution of 0.1% (w/v) bovine serum albumin (BSA), obtained from Sigma-Aldrich, by centrifugation (at 1400 *g* for 1 min). These were used for cell culturing, PCR, and other operations. The aperture of this MMV was sealed with silicone tape and incubated overnight at 4°C. After incubation, the solution was removed by centrifugation (O-mode), and the MMV was dried at room temperature for 1 h.

### Replica formation

A typical example of replica formation is shown in Additional file 4: Figure S3, where a cherry blossom pattern was formed on MMV using two colors (Elements 1 and 2). Element 1 was charged in E1-MMV using a #1 filter with a thickness of 0.3 mm and a hole diameter of 0.5 mm for expression of the cherry blossom pattern. E2-MMV contained Element 2 in the complementary pattern of Element 1. Here, Element 1 was a DNA (Aβ 42-binding peptide gene)-containing PCR mixture, whereas Element 2 contained no DNA. The PCR mixture comprised 1× FB I buffer (Takara), 250 μM dNTP mixture, 0.04 μM CA primer (5′-CAACACACCACCCACCCAAC-3′), and 0.025 U/μL SpeedSTAR HS DNA polymerase (Takara). Both E1- and E2-MMVs were subjected to PCR and then combined to generate the original MMV. This original MMV was used for the replica formation by a stamping method wherein a spacer with a thickness of 0.8 mm and hole diameter of 0.6 mm (Finetech) was interposed between the original (donor) and the acceptor MMVs. The centrifugal transfer (1300 *g* for 1 min) from the donor to the acceptor MMV was reversed just after the first transfer, resulting in the generation of an MMV in which a very small aliquot remained. This aliquot can serve as a seed for PCR amplification (DNA), thus reproducing a replica.

A replica of cells is generated by a similar operation as described above. For this demonstration, GFP-plasmid-harboring *Escherichia coli* cells were used and cultured in LB broth [0.5% (w/v) yeast extract, 1% (w/v) tryptone, and 1% (w/v) NaCl with 0.1 μg/μL ampicillin and 1 mM IPTG]. Here, Element 1 corresponds to GFP-harboring *E. coli* and Element 2 to LB medium without cells.

### MMV setup in a conventional thermocycler

In a conventional thermocycler (Bio-Rad C1000 Touch), for better heat conduction, a copper plate was inserted between the PCR block and the MMV chip. In addition, MMV was sealed with silicone tape, occluded with aluminum foil, and installed in the thermocycler. Silicone rubber was used to closely pack the MMV to protect against pressure.

### Imaging of MMV reaction products

The staining of PCR products with SYBR Green I (Lonza) was performed using a shallow well MMV (0.1 μL/well) according to the manufacturer’s instructions. Before the transfer (1300 *g* for 1 min) of the dye from the shallow MMV to the PCR-product-containing MMV, the contents of the latter were reduced in advance by dry air-driven evaporation (lid was left open on a clean bench for few minutes) to make space for the solution to be transferred. Further, MMV with stained DNA was monitored using a fluoroimager (Molecular Imager FX, Bio-Rad). MMV with *E. coli* cells was visualized by FITC staining and monitored using the same fluoroimager.

### Lysozyme crystallization

We applied the 2^N^ method to test 1024 different conditions for lysozyme crystallization (Additional file 11: Figure S10 and Additional file 12). Lyophilized powder of lysozyme (Sigma-Aldrich) was dissolved in 5 mM MES buffer (pH 6.6) [final concentration of 28% (w/v), final volume of 1 mL]. This solution was carefully vortexed to eliminate any visible flocs of lysozyme and filtered through a membrane filter (0.2-μm pore size), and the supernatant was collected for use in the crystallization experiment. Stepwise different pH values were generated with filter spacers of F0, F1, F2, F3, and F4 (Figure 2a; S-mode operation) charged with approximately 40 nL of 1 M sodium citrate buffer (pH 2.9), 1 M sodium acetate buffer (pH 3.9), 1 M MES-NaOH (pH 6.6), 1 M Tris–HCl (pH 8.5), and 1 M glycine buffer (pH 9.6), respectively. For the condition of a different ionic strength, filter spacers of F5, F6, F7, F8, and F9 were charged with approximately 40 nL of 1 M, 2 M, 3 M, 4 M, and 5 M NaCl solution, respectively. To equalize the volume of solution added to each well, distilled water was added to the wells not charged with any of F0–F9 using the templates $\left(\bar{F0}-\bar{F9}\right)$ complementary to F0–F9 (the well position of F_i_ is not a well in $\bar{\mathrm{Fi}}$ and inversely, a nonwell position of F_i_ is assigned as a well in $\bar{\mathrm{Fi}}\text{;}$*i* = 0, 1, 2). Finally, lysozyme solution (280 mg/mL) was added (approximately 40 nL) to the wells. The crystallization was performed by leaving the MMV in the middle of a closed container (10 cm × 10 cm × 10 cm) with its interior soaked with 500 mL of 2 M ammonium sulfate at 20°C for one day to one week. Each well was monitored with an inverted microscope (IM; Olympus, Tokyo, Japan).

### NGS-non-dependent microbiome analysis

#### Sample collection

A saliva sample was collected from one of the authors (with their consent), by themselves 5 h after brushing and with no intake of food in this period. This sample-collection was performed with the permission of the Saitama University Ethical Committee on Human-related Research. For sample collection, two small, sterile sponge pieces were kept bitten in the mouth for 5 min and then dipped in 1 mL of Dulbecco’s phosphate buffered saline [DPBS (−)] (pH 7.4) (Wako Pure Chemical Industries, Ltd.). The saliva was squeezed out of the sponge pieces and was dissolved in PBS and subjected to centrifugation for 5 min at 1470 *g*. The supernatant was discarded; the pellet was dissolved in 200 μL of DPBS (−) buffer and stored in 13% glycerol at −80°C.

#### Deflocculation and cell counting

To dissolve the flocs of cells, 1 μL of the oral sample was treated with 9 μl of DPBS (−) buffer and 200 pg/μL proteinase K. This solution was sonicated for 40 s, incubated at 55°C for 1 h and at 95°C for 5 min, and then again sonicated for 40 s. This deflocculation-treated solution was subjected to staining with 10000× diluted SYBR gold solution, and cells were counted using a Neubauer improved cell counting chamber.

#### Single-cell DNA extraction

The oral microbes were suspended in bacterial cell lysis reaction buffer containing 10 mM Tris–HCl (pH 8.0), 0.025% BSA, 0.10% Tween 20 (Wako Pure Chemical Industries, Ltd.), and 0.75% polyethylene glycol 8000 (PEG8000, Sigma-Aldrich) to obtain a concentration of 300 cells/700 μL and to lyse the cells. Moreover, achromopeptidase [0.004% (w/v), Wako Pure Chemical Industries, Ltd.] solution was added to this extraction mixture. The resulting solution was immediately charged into a shallow well-type MMV (0.14 μL/well capacity) and then transferred to a full volume-type MMV (0.5 μL/well capacity) by Z-mode transfer and incubated at 37°C for 1 h and then at 95°C for 10 min.

#### Single-cell random PCR

PCR mixture containing 200 μM dNTPs (N = G, A, T, or C), 0.7 μM primer pfM 19 (5′-CAGGGCGCGTAC-3′), 10 mM Tris–HCl (pH 8.3), 50 mM KCl, 1.5 mM MgCl_2_, 0.03 U/μL Taq DNA polymerase (Takara), and an additional solution of 0.025% BSA, 0.75% PEG 8000, and 0.1% Tween 20 were loaded into the MMV treated with bacterial cell lysis reaction buffer. A dodecamer primer, pfM 19, was used for random PCR. The MMV chip was sealed tightly with silicone tape and aluminum foil to prevent evaporation of the reaction mixture during PCR.

To minimize the amplification of undesirable DNAs such as primer dimers, the PCR program comprising the two following series was chosen: *i*) 15 cycles of denaturation at 94°C for 30 s, annealing at 26°C for 60 s, and extension at 72°C for 60 s and *ii*) 20 cycles of denaturation at 94°C for 30 s, annealing at 60°C for 60 s, and extension at 72°C for 60 s. PCR was performed using an MMV PCR machine (Lifetech) or a conventional thermocycler (Bio-Rad C1000 Touch).

After PCR, the MMV was stained with SYBR green I and monitored using a fluoroimager. MMV wells showing high fluorescence (dark pixels) were selected for scale-up PCR and transferred to a 96-well PCR plate. For scale-up PCR, a PCR mixture that composed of 200 μM dNTPs (N = G, A, T, or C), 0.7 μM primer pfM 19, 10 mM Tris–HCl (pH 8.3), 50 mM KCl, 1.5 mM MgCl_2_, and 0.02 U/μL Taq DNA polymerase (Takara) was used. Samples were amplified by 15 cycles of denaturation at 94°C for 30 s, annealing at 26°C for 60 s, and extension at 72°C for 60 s.

#### PCR product analysis

Random PCR products were analyzed using μTGGE, which can extract sequence-specific information of double-stranded DNA without sequencing (Figure 4b and Additional file 12). Each profile was assigned genome-specific feature points called *spiddos*[40], and *PaSS* (pattern similarity score) was calculated (Additional file 12). The genome distance *d*_*G*_, defined as the value of 1 − *PaSS*, was subjected to clustering analysis to construct a phylogenetic tree using the neighbor-joining method with Phylip 3.69 and MEGA 5.1 software.

## Abbreviations

MMV: Microarray with manageable volumes; GP: Genome profiling; μTGGE: Micro-temperature gradient gel electrophoresis; *Spiddos*: Species identification dots; *PaSS*: Pattern similarity score; NGS: Next generation sequencing; NNMA: NGS-non-dependent microbiome analysis; ccgf: Commonly conserved genetic fragment; C2C: Cell-to-cell; D2D: DNA-to-DNA; C2D: Cell-to-DNA; D2P: DNA-to-peptide/protein; C2D2P: Cell-to-DNA-to-peptide/protein; iPS: Induced pluripotent stem cell.

## Competing interests

Nine of the authors are affiliated with four commercial organizations: SF, MiSa and KK with Janusys Corporation, MaS, KS and OT with Lifetech Co., Ltd., ST and TW with Enplas Corporation, and HY with Finetech Corporation. The corresponding author (KN) was financially supported in 2010 by Lifetech Co. Two of the authors (KK and NN) hold shares of Janusys Corporation. The following patents related to this paper are applied by some of the authors: Patent Application Publication No. 2013–195370 (YK, SA, ST, HY, OT, KN). Patent Application No. 2013–177214 (YK, SF, HY, KK, OT, KN). The authors declare that they have no other competing interests.

## Authors’ contributions

HS performed the NNMA experiment and wrote/co-edited the manuscript. YK and SF developed the MMV basic handling technologies and performed the lysozyme crystallization and replica generation experiments, respectively. SN, MiSa, and SA initiated and developed the NNMA, MMV-cell-based assay, and MMV-based peptide screening experiments, respectively. MaS, KS, and OT chiefly contributed to manufacturing the MMV robot. ST, TW, and HY devised the MMV chips and accessories. RK and TK performed the confirmation experiments. HA discussed and performed essential calculations. MiSu and NN discussed and directed this study. KA and HU established the MMV-fluorescence detection. KK and OT (again) contributed to jointly organizing, discussing, and directing this joint research. KN conceived, organized, and directed the whole research and edited and revised the manuscript. All authors read and approved the final manuscript.

## Supplementary Material

Additional file 1: Table S1 Unit technologies and accessories of the MMV system. Click here for file

Additional file 2: Figure S1 Transfer of solution in a square (thin filter) to MMV wells (S-mode operation). Here, the holes of a thin filter act as squares. The filling process is explained in the Figure 1c legend. Click here for file

Additional file 3: Figure S2 Robotic transfer of solution to an MMV well (P-mode operation). A robot developed for P-mode transfer in MMV operations. Both MMV-to-MMV and MMV-to-microplate or other transfers can be performed by this machine. This robot (manufactured by Lifetech) comprises robotic dispenser arms (1), three platforms [one for microplate (2) and two for MMVs (5, 6)], a tip or syringe stand (3), a container to discard used tips (4), and a tip-position sensor (7). The whole system is controlled by a computer (not shown). The tip-position sensor is required to adjust and control the fine (10 μm or less) 3D positions of tips. Click here for file

Additional file 4: Figure S3 Generation of MMV replicas. **(a)** An example of preparing an original MMV made of two types of wells (Elements 1 and 2) in a specific pattern. Element 1-containing MMV (E1-MMV) was prepared using a specific pattern filter, and similarly, Element 2-containing MMV (E2-MMV) was prepared with a pattern complementary to that of E1-MMV. These two MMVs were combined by transferring the contents of E1-MMV to E2-MMV. **(b)** The contents of the original MMV were transferred to the vacant acceptor MMV by centrifugation and then reversed as shown, leaving a small amount of solution (seed) in each well of replica MMV. These seeds may be DNA or cells depending on the type of replica formation. **(c)** Picture of wells containing tiny droplets. **(d)** Actual microscopic image of droplet containing MMV wells. Click here for file

Additional file 5: Figure S4 Actual checker-pattern experiment for verification of MMV solution transfer operation (Z-mode). Methylene blue dye solution is charged into a checker-pattern packing spacer (filled and empty wells alternatively) on a small area of the donor MMV and transferred to the acceptor MMV by Z-mode transfer, thus transferring solution only to corresponding wells without cross-contamination. Click here for file

Additional file 6: Figure S5 Ejection of MMV contents (O-mode operation: washing). **(a)** View of a polycarbonate MMV. **(b)** Schematic representation of solution discharge from an MMV chip. An MMV chip covered with a sheet of filter and tissue paper is centrifuged to eject the contained solutions and can be repeatedly washed by the same process. Click here for file

Additional file 7: Figure S6 Division of solutions in MMV (X-mode operation). A filter patterned with two small holes per well was attached to an empty acceptor MMV and was tightly bound to a filled (donor) MMV. These sets of MMVs were subjected to centrifugation directed parallel to the MMV surface. On centrifugation, the liquids were divided into two portions in the facing donor and acceptor MMV wells. Click here for file

Additional file 8: Figure S7 Magnetic bead recovery from solutions (M-mode operation). Magnetic beads bound to the desired molecules can be recovered by the following steps. Mixing of beads-containing solution can be performed by sealing the solution in MMV with silicone tape and the alternative attractive force of magnets. The recovered magnetic beads bind the desired molecule ‘A’ on their surface via the ‘anti-A’ molecule directly bound to the bead. Click here for file

Additional file 9: Figure S8 Magnetizable bead-assisted division of liquid (M-mode operation). Using packing with holes smaller than the bead diameter, the solution in the 70% volume of MMV can be retained during centrifugation [step b) to c)]. Click here for file

Additional file 10: Figure S9 Pipette-dependent transfer of solution in an MMV well (P-mode operation). (a) Image of a pipette, tip, and MMV chip. (b) Close-up view of manual pipette operation along MMV wells. Click here for file

Additional file 11: Figure S10 Generation of diverse conditions. N-times of 2 state (2^N^) method. Each well of an MMV can be uniquely assigned by a binary number composed of N-bits (here, N = 4). Here, “0” or “1” at each position of a binary number corresponds to the absence or presence, respectively, of a specific component input using the corresponding plate. Thus, “0000” means the absence and “1111” the presence of all four components. Similarly, “0101” implies the presence of only the second and fourth components. Click here for file

Additional file 12 2^N^ method, genome profiling, μTGGE, sequencing analysis, and peptide aptamer selection: panning on microarray MMV (POMM) methods are described in detail. Click here for file

Additional file 13: Figure S11 Phase-diagram-like presentation of lysozyme crystals. **(a)** Different conditions composed of pH (2.9–9.6) and ionic strength (NaCl: 0–1.5 M) generated different types of lysozyme crystals. Each shape of crystal in MMV wells is depicted in different colors. Four main types of crystals (large and small tetragonal crystals and microcrystalline and needle-like crystals) were observed. **(b)** Microscopic image of a crystal (0.65 mm in length) obtained by the lysozyme crystallization reproducibility experiment (at 50 μL scale) performed under one of the conditions generated in the MMV chip, i.e., 0.6 M NaCl and pH 8.6. The scale bar is 100 μm. Click here for file

Additional file 14: Figure S12 Original genome profiles obtained from random PCR-successful microbial samples appearing in the MMV-PCR experiment. Samples that have been successfully processed up to clustering analysis (Figure 4d in text) are shown. Sample feature points (pre-*spiddos*) and internal reference points are indicated by red and yellow dots, respectively. Click here for file

Additional file 15: Table S2 BLASTN-aided tentative assignment of species for each DNA band in the NNMA experiment. Click here for file

Additional file 16: Table S3 Basic data obtained for three trials of the NNMA experiments. Click here for file

Additional file 17: Figure S13 Well-to-well transfer by nanoliter dispenser robot. Transfer of 0.1 μL volume of solution from upper donor wells (A, B, and C) to lower acceptor wells (A′, B′, and C′) by robotic transfer in the same MMV. In this experiment, around 5% solution (0.005 μL) is left after transfer as shown in the crescent shape (upper row) due to the difficulty of withdrawing all the solution. Click here for file

Additional file 18: Figure S14 Micro-high-throughput screening (μ-HTS) of Aβ-binding peptides by panning on microarray MMV (POMM). A DNA library of candidate sequences was transferred to the MMV. Each candidate sequence contained the T7 promoter region and was tagged with 3× FLAG. MMV PCR was performed, and MMV was replicated. The replica MMV was stored (at −20°C) for future replication and screening experiments. For subsequent steps, the original MMV was used. DNA sequences were *in vitro* transcribed and translated (IVT) (see Additional file 12). Selectively Aβ 42-binding peptides were extracted using streptavidin magnetic beads and biotin-conjugated Aβ 42. MMV was thoroughly washed to eliminate everything except binding peptides. FITC-labeled anti-FLAG antibody solution was added, MMV was washed, and peptides were released by proteinase K treatment. MMV was visualized in the detection unit (CCD camera or TRF unit or laser scanner with FITC filter). In addition, MMV wells stained with FITC indicated the presence of Aβ-42 binding peptides, resulting in fluorescence, whereas in the absence of Aβ-binding peptide, FITC-labeled anti-FLAG antibody did not bind and was washed out in the initial steps, thus resulting in no fluorescence. Click here for file

Additional file 19: Figure S15 Deflocculation of an oral microbiome sample. **(a)** Microscopic view of SYBR gold-stained negative (PBS buffer only), positive (oral microbiome sample without treatment), heat (positive sample with heat treatment only), and proteinase K (positive sample with proteinase K treatment only) samples. **(b)** Phase-contrast microscopic images of an oral microbiome sample treated with proteinase K only and with both proteinase K and sonication (for 1 min). The scale bar is 50 μm for both **(a)** and **(b)**. The ratio of single to flocculated cells was approximately 100:1. Click here for file

Additional file 20: Figure S16 A trial semiconductor-based apparatus for the evaluation of solution conductivity in MMV wells. The conductivity of the solution in a particular well can be selectively monitored by laser-light illumination through light-transparent semiconductor ITO and photoconductive polymer membrane. The figure shows the redox reaction occurring on the surface of the photoconductive polymer membrane. Click here for file

Additional file 21: Table S4 Tentative cost comparison of the reagents required in MMV and a 96-well microplate (for 1000 reactions). **(a)** Cost comparison for a multistep function-based screening experiment. **(b)** Cost comparison for the experiment of apoptosis detection in HeLa cells. The prices were taken from those in 2012 in Japan for both tables **(a)** and **(b)**. Click here for file
