# Supplementary material for: Establishment of a reborn MMV-microarray technology: realization of microbiome analysis and other hitherto inaccessible technologies
Source: BMC Biotechnol. 2014 Aug 21;14:78. doi: 10.1186/1472-6750-14-78 (PMC4153446; doi:10.1186/1472-6750-14-78)
Supplement: Additional file 1: Table S1 — Unit technologies and accessories of the MMV system. [file 1472-6750-14-78-S1.docx]

**Additional file 1: Table S1.** Unit technologies and accessories of the MMV system.

| **Unit Technologies** | | **Content** | **Category** | **Comments** | **Level Obtained** |
| --- | --- | --- | --- | --- | --- |
| **Manipulation** | Dump transfer | Well-to-well vertical transfer of solution from the donor to acceptor MMV using a spacer. | Simple manipulation | High transfer accuracy | Successfully achieved |
|  | Selective transfer | MMV-to-MMV selective transfer of solutions in specific wells using either of the following:  **1.** Packing (different patterns of holes) or  **2.** MMV dispenser system | In some instances, automation is required. | Aids generation of 1,024 different conditions. | Successfully achieved |
|  | Addition/mixing | Addition and mixing using packing of different patterns of holes and thickness.  In addition, mixing by vigorous moving of small steel beads under a magnetic force. | Relatively complex manipulation |  | Successfully achieved |
|  | Division | Division of solution between two MMVs using the following:  **1.** By mainly specific filter or packing or stainless steel beads mediated well-to-well vertical (Z-type) or horizontal (X-type) transfer of a fraction of the solution.  **2.** Partially by MMV dispenser | Simple manipulation, but careful handling required. | Transfer rate needs to be controlled in some instances. | Method successfully developed, but optimization is required in some cases. |
|  | PCR | Isolation of cells, DNA extraction, and amplification of DNA, all in a single MMV chip. | Simple manipulation | MMV chip found to be compatible to the conventional thermocycler. | Successfully achieved (up to single molecule/0.5 µl level) |
|  | Coating | To prevent biofouling of molecules, MMV surface was coated using the following:  **1.** BSA (spin coating),  **2.** Silanization solution (coating by vapor deposition),  **3.** mPEG Thiol (spin coating) | Simple manipulation | BSA coating found to be most suitable. | Successfully developed and tested |
|  | Evaporation | Exposing MMV in a laminar air flow to evaporate excess solution for the prevention of cross-contamination and for further addition of chemicals. | Simple manipulation | Control of evaporation rate in each well needs to be achieved. | Successfully achieved |
|  | Pipetting | Recovery of solutions from MMV using the following:  **1.** MMV dispenser system,  **2.** Pipetting under a microscope | Slightly complex manipulation | Manual handling requires care and time. | Successfully developed and tested |
|  | Culture | Addition of media inoculated by cells of either of the following:  **1.** Bacteria,  **2.** Fungi,  **3.** Animal cells,  followed by incubation and parallel observation of growth under different conditions. | Simple manipulation | Single cell culture is possible without traditional laborious colony-picking method. | Successfully achieved |
|  | Replica | Partial well-to-well transfer and back transfer of solutions leading to the distribution of the original solution to each well of the original and the replica MMVs (see **Methods**)  **1.** Replica of DNA,  **2.** Replica of cells | Simple manipulation | Useful in culture collection and library stock preparation. | Successfully achieved |
|  | Storage | Preservation of MMV at the following:  **1.** 4 °C,  **2.** −20 °C,  **3.** −80 °C,  **4.** Liquid nitrogen | Simple handling |  | Successfully achieved |
| **Detection** | Fluorescence | Staining of reaction products using fluorescent dyes (SYBR green I, SYBR gold, etc.), or detection of sample-specific fluorescence using a fluorescence microscope or fluoroimager. | Simple manipulation |  | Successfully achieved |
|  | Microscope | Visualization of MMV wells under optical microscope to observe sample charging and manipulation. | Simple handling |  | Successfully achieved |
|  | 4SR | Direct 3D electrophoresis of reaction products in each well of MMV using stacking on layers of sliced gels. | Relatively simple, multiparallel, and large-scale analysis of DNA/RNA/proteins/peptide. | Needs to be tested in polycarbonate MMV. | Successfully achieved (in polyacrylamide MMV) |
| **Accessories** | Frame | Silicon/urethane frame confines edges of MMV and helps in initial addition of solution. | Transfer accessory |  | Currently in use |
|  | Packing | Silicon/urethane packing for addition of solution to MMV and generation of multiple conditions. | Transfer accessory |  | Currently in use |
|  | Filter/spacer | Aids well-to-well transfer of solution from the donor to acceptor MMVs. Mainly constructed of silicon/urethane. | Transfer accessory |  | Currently in use |
|  | Beads and magnet-embedded centrifugal stand | 0.5-mm stainless steel beads and magnet were used for mixing the solution in MMV. | Transfer and centrifugation accessory | Stand was built in-house | Currently in use |
|  | Plastic case | To keep MMV in centrifuge basket for general transfer processes. | Basic accessory (centrifugation, transfer, storage, etc.) |  | Currently in use |
|  | Centrifuge flat bucket | For centrifugation of MMV. | Centrifugation accessory |  | Currently in use |
|  | Horizontal (X-mode) transfer MMV stand | To keep the donor and acceptor MMVs together and prevent leakage during centrifugal X-mode transfer. | Transfer and centrifugation accessory | Built in-house | Currently in use |
|  | Silicone tape | Used for sealing MMV and prevention of evaporation. | Basic accessory |  | Currently in use |
|  | MMV holder | To set MMV in MMV PCR machine. | PCR accessory |  | Currently in use |
|  | Silicone rubber and copper plate | For MMV PCR in conventional thermocycler. | PCR accessory |  | Currently in use |
