# Supplementary figures and images for: Establishment of a reborn MMV-microarray technology: realization of microbiome analysis and other hitherto inaccessible technologies
Source: BMC Biotechnol. 2014 Aug 21;14:78. doi: 10.1186/1472-6750-14-78 (PMC4153446; doi:10.1186/1472-6750-14-78)

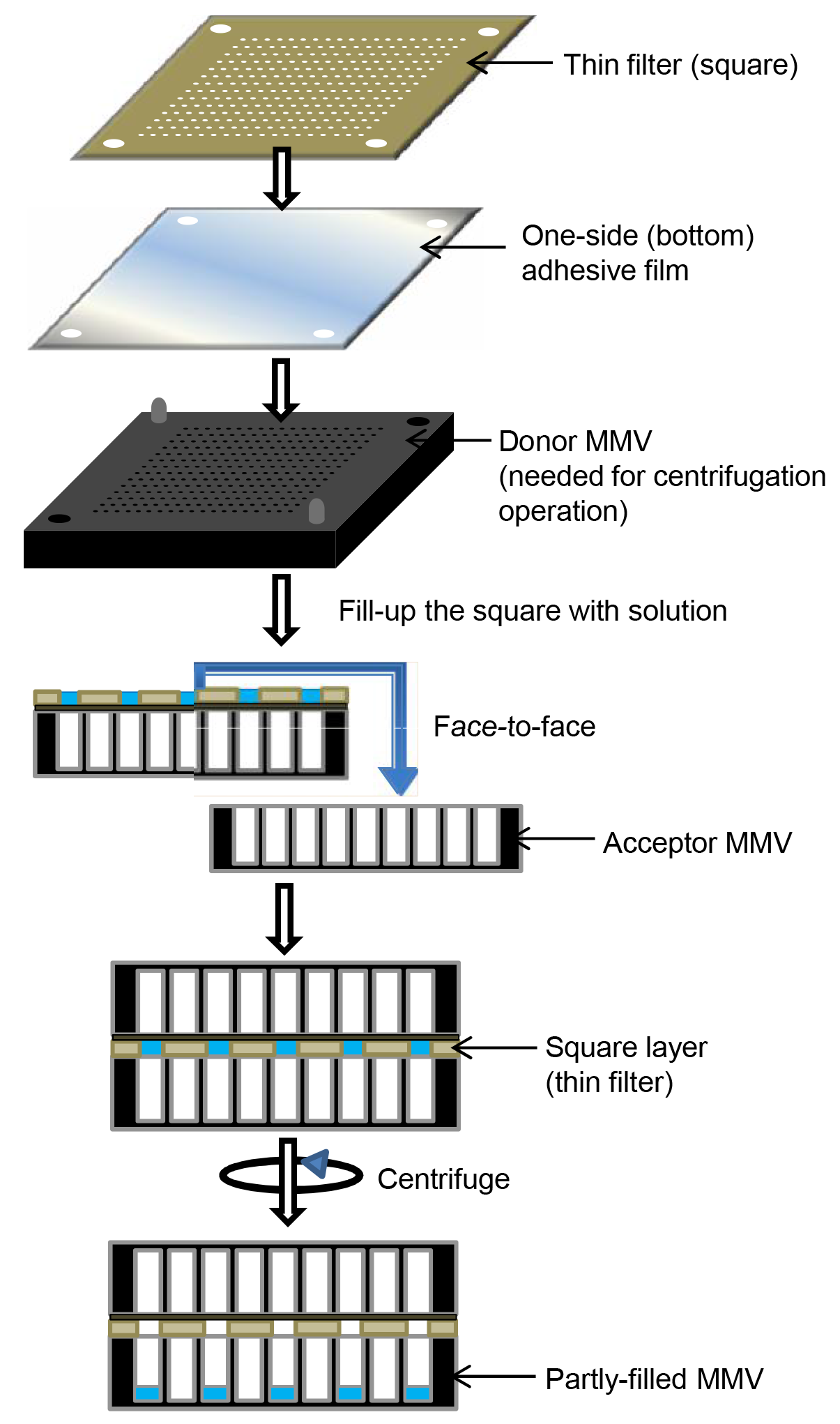

Supplement: Additional file 2: Figure S1 — Transfer of solution in a square (thin filter) to MMV wells (S-mode operation). Here, the holes of a thin filter act as squares. The filling process is explained in the Figure 1c legend. [file 1472-6750-14-78-S2.tiff]

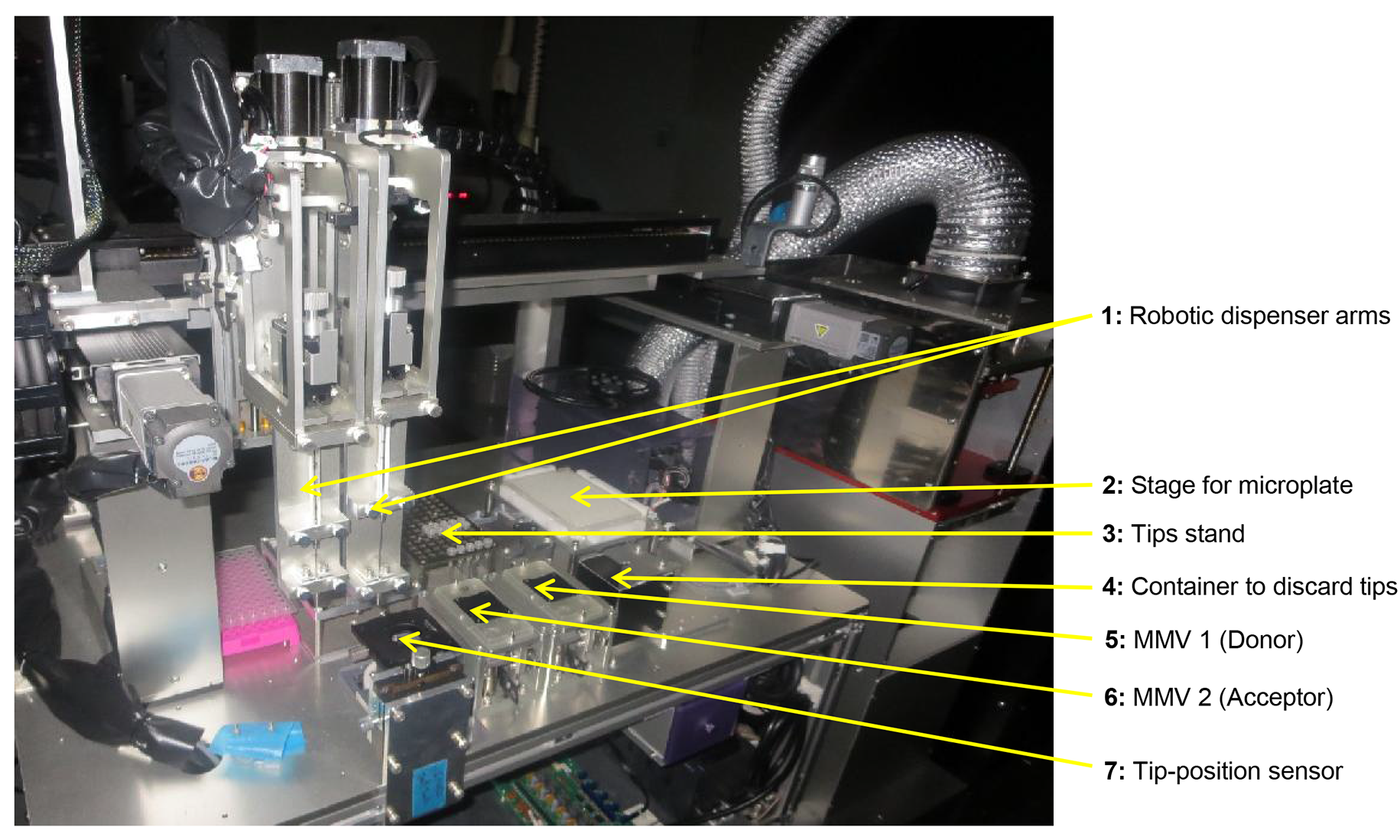

Supplement: Additional file 3: Figure S2 — Robotic transfer of solution to an MMV well (P-mode operation). A robot developed for P-mode transfer in MMV operations. Both MMV-to-MMV and MMV-to-microplate or other transfers can be performed by this machine. This robot (manufactured by Lifetech) comprises robotic dispenser arms (1), three platforms [one for microplate (2) and two for MMVs (5, 6)], a tip or syringe stand (3), a container to discard used tips (4), and a tip-position sensor (7). The whole system is controlled by a computer (not shown). The tip-position sensor is required to adjust and control the fine (10 μm or less) 3D positions of tips. [file 1472-6750-14-78-S3.tiff]

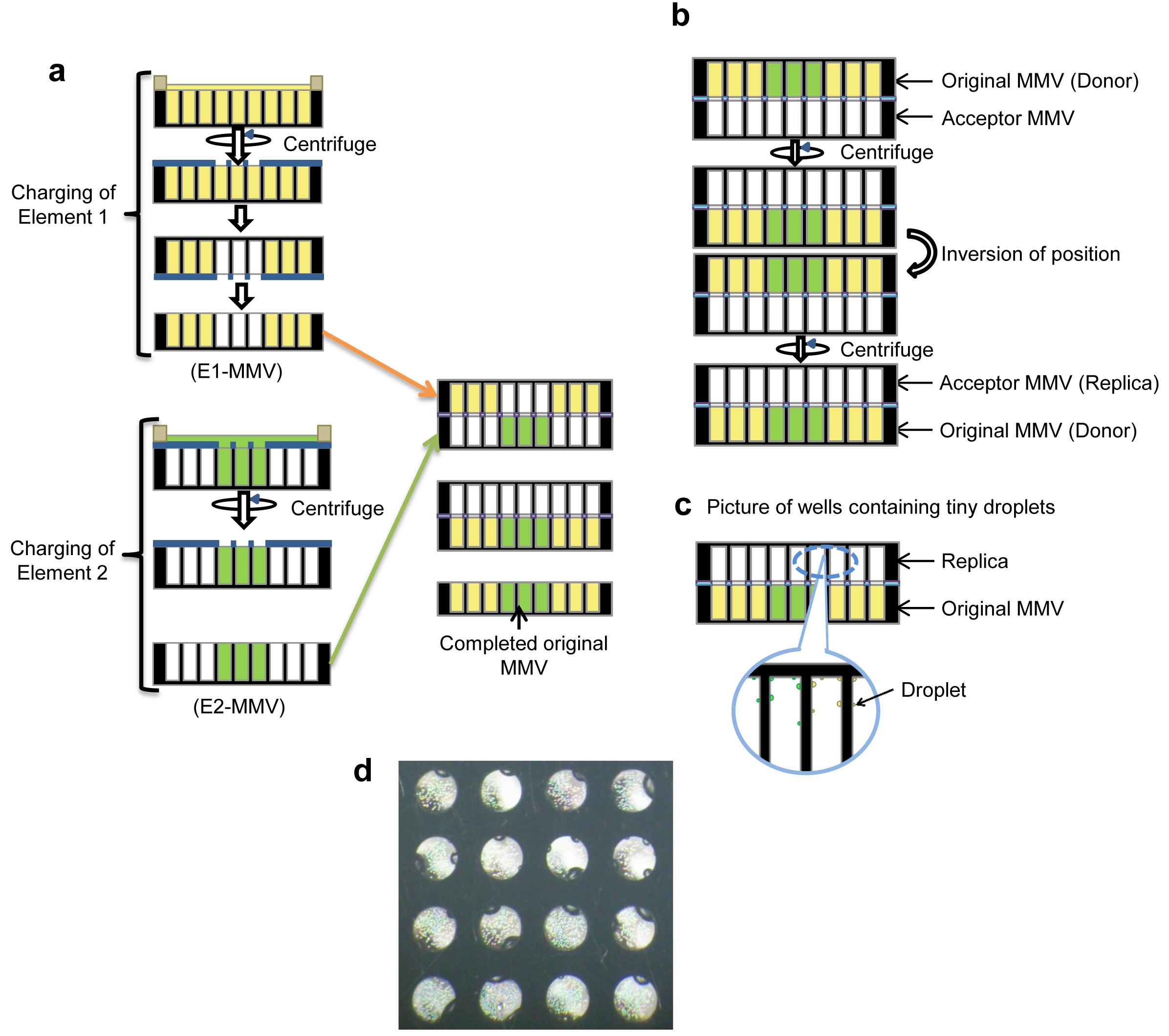

Supplement: Additional file 4: Figure S3 — Generation of MMV replicas. (a) An example of preparing an original MMV made of two types of wells (Elements 1 and 2) in a specific pattern. Element 1-containing MMV (E1-MMV) was prepared using a specific pattern filter, and similarly, Element 2-containing MMV (E2-MMV) was prepared with a pattern complementary to that of E1-MMV. These two MMVs were combined by transferring the contents of E1-MMV to E2-MMV. (b) The contents of the original MMV were transferred to the vacant acceptor MMV by centrifugation and then reversed as shown, leaving a small amount of solution (seed) in each well of replica MMV. These seeds may be DNA or cells depending on the type of replica formation. (c) Picture of wells containing tiny droplets. (d) Actual microscopic image of droplet containing MMV wells. [file 1472-6750-14-78-S4.tiff]

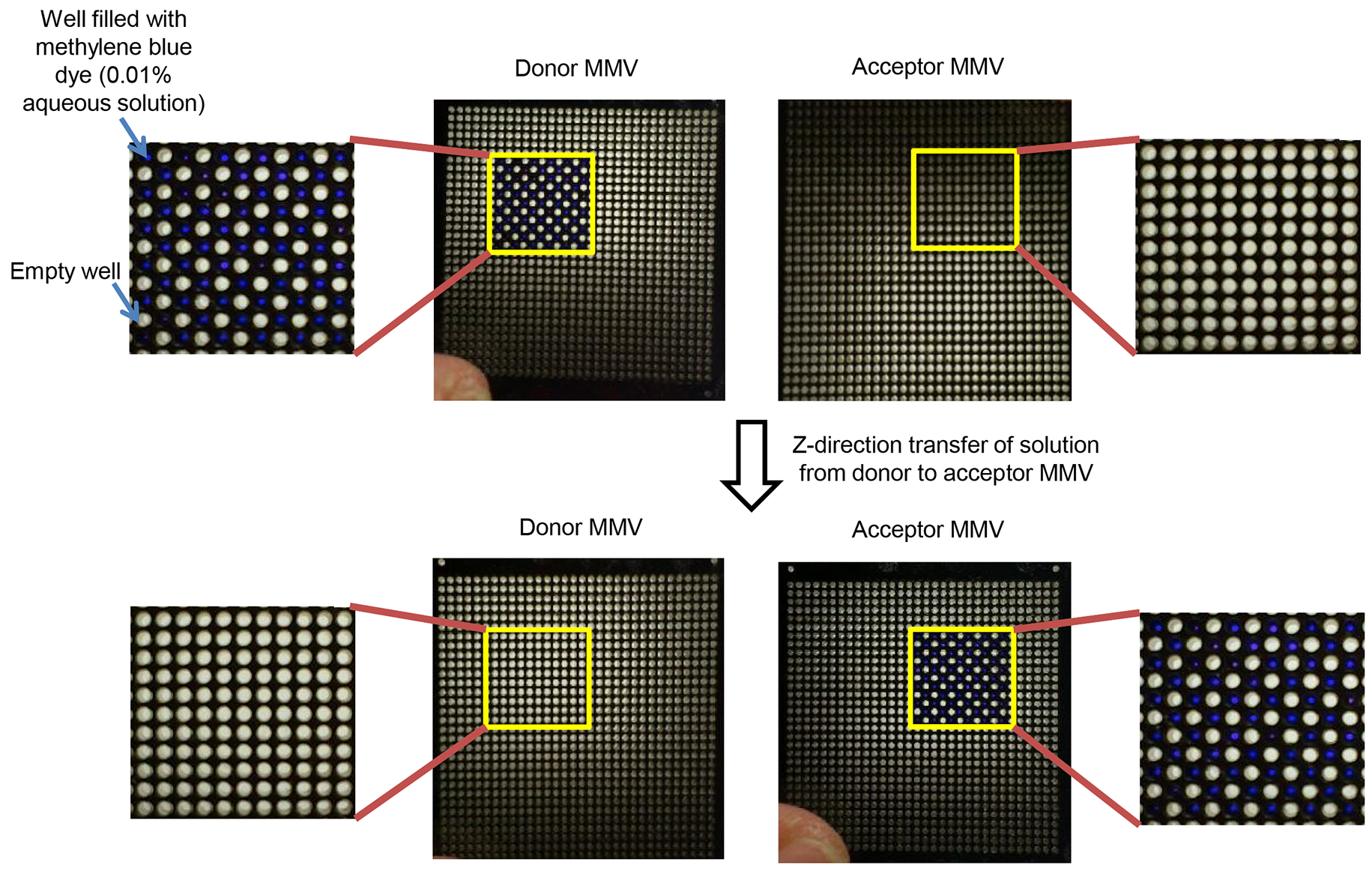

Supplement: Additional file 5: Figure S4 — Actual checker-pattern experiment for verification of MMV solution transfer operation (Z-mode). Methylene blue dye solution is charged into a checker-pattern packing spacer (filled and empty wells alternatively) on a small area of the donor MMV and transferred to the acceptor MMV by Z-mode transfer, thus transferring solution only to corresponding wells without cross-contamination. [file 1472-6750-14-78-S5.tiff]

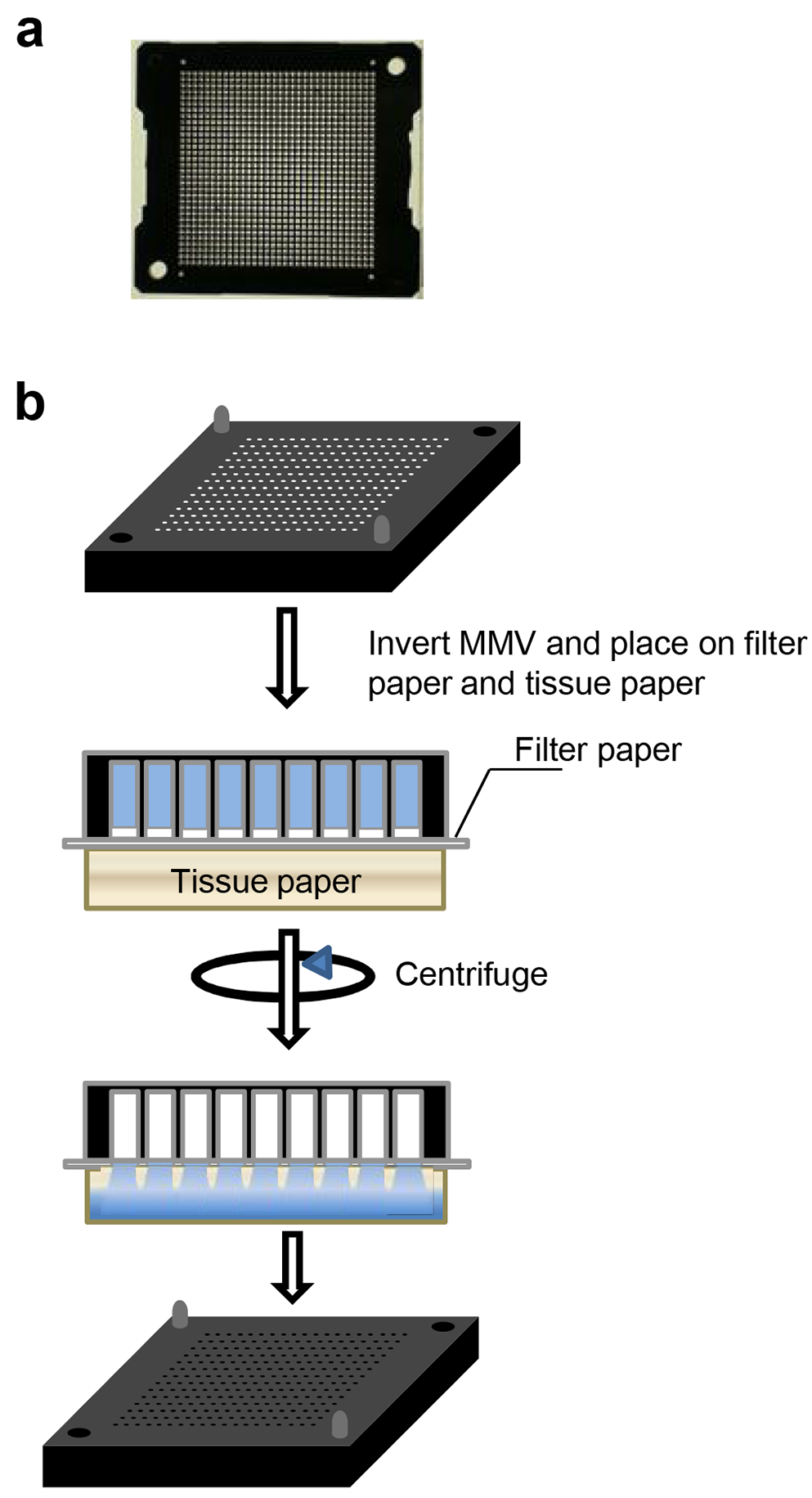

Supplement: Additional file 6: Figure S5 — Ejection of MMV contents (O-mode operation: washing). (a) View of a polycarbonate MMV. (b) Schematic representation of solution discharge from an MMV chip. An MMV chip covered with a sheet of filter and tissue paper is centrifuged to eject the contained solutions and can be repeatedly washed by the same process. [file 1472-6750-14-78-S6.tiff]

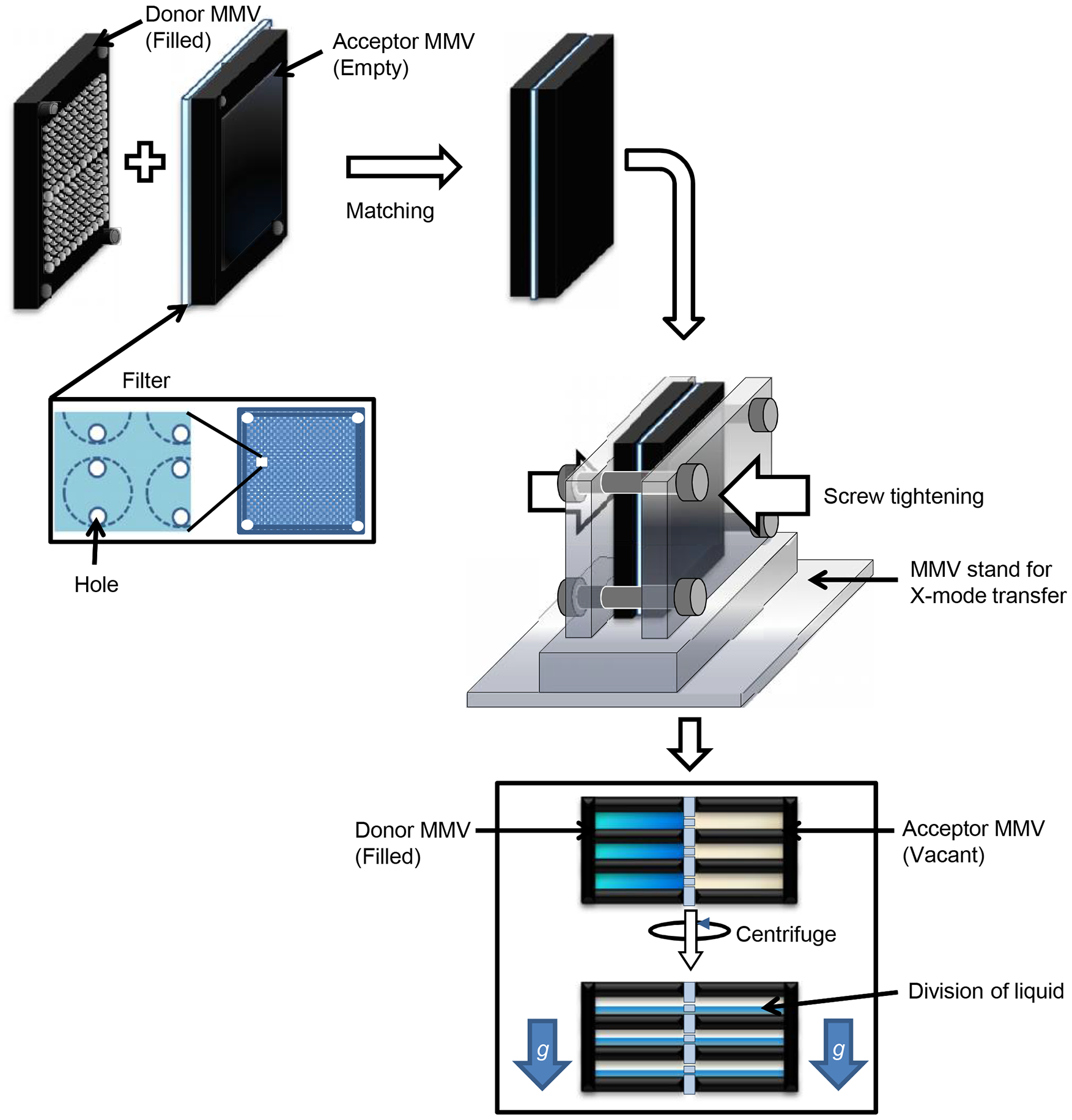

Supplement: Additional file 7: Figure S6 — Division of solutions in MMV (X-mode operation). A filter patterned with two small holes per well was attached to an empty acceptor MMV and was tightly bound to a filled (donor) MMV. These sets of MMVs were subjected to centrifugation directed parallel to the MMV surface. On centrifugation, the liquids were divided into two portions in the facing donor and acceptor MMV wells. [file 1472-6750-14-78-S7.tiff]

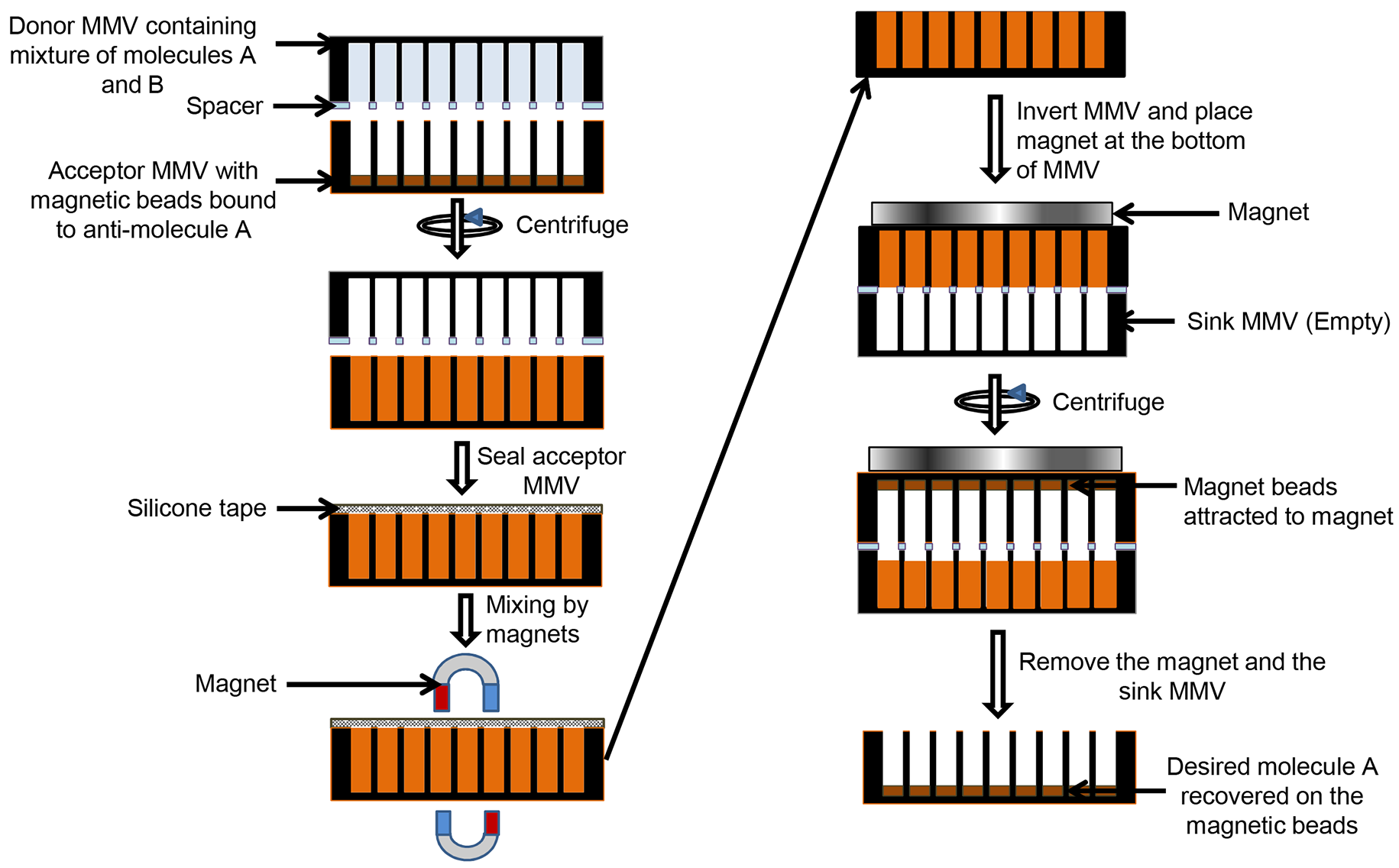

Supplement: Additional file 8: Figure S7 — Magnetic bead recovery from solutions (M-mode operation). Magnetic beads bound to the desired molecules can be recovered by the following steps. Mixing of beads-containing solution can be performed by sealing the solution in MMV with silicone tape and the alternative attractive force of magnets. The recovered magnetic beads bind the desired molecule ‘A’ on their surface via the ‘anti-A’ molecule directly bound to the bead. [file 1472-6750-14-78-S8.tiff]

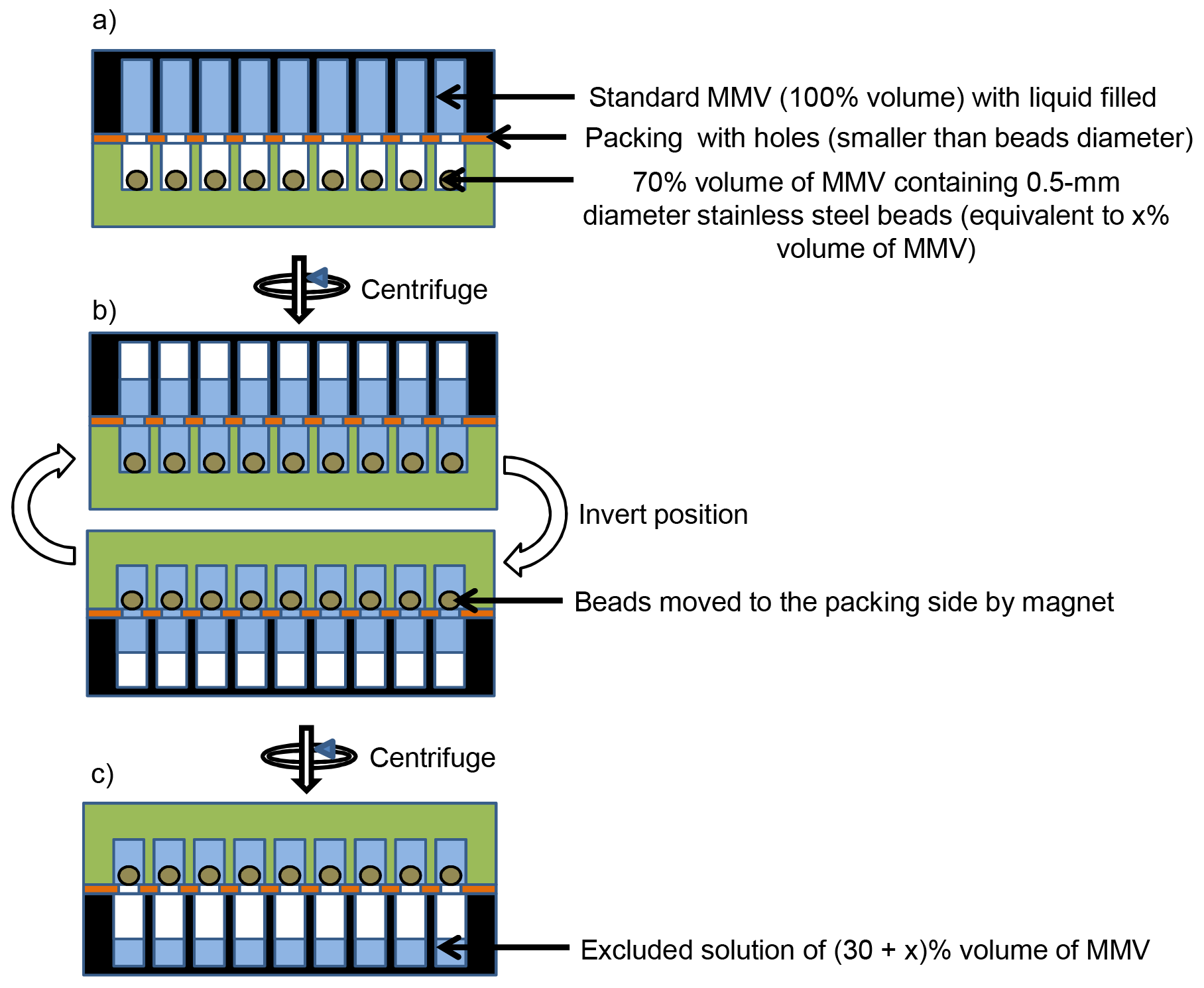

Supplement: Additional file 9: Figure S8 — Magnetizable bead-assisted division of liquid (M-mode operation). Using packing with holes smaller than the bead diameter, the solution in the 70% volume of MMV can be retained during centrifugation [step b) to c)]. [file 1472-6750-14-78-S9.tiff]

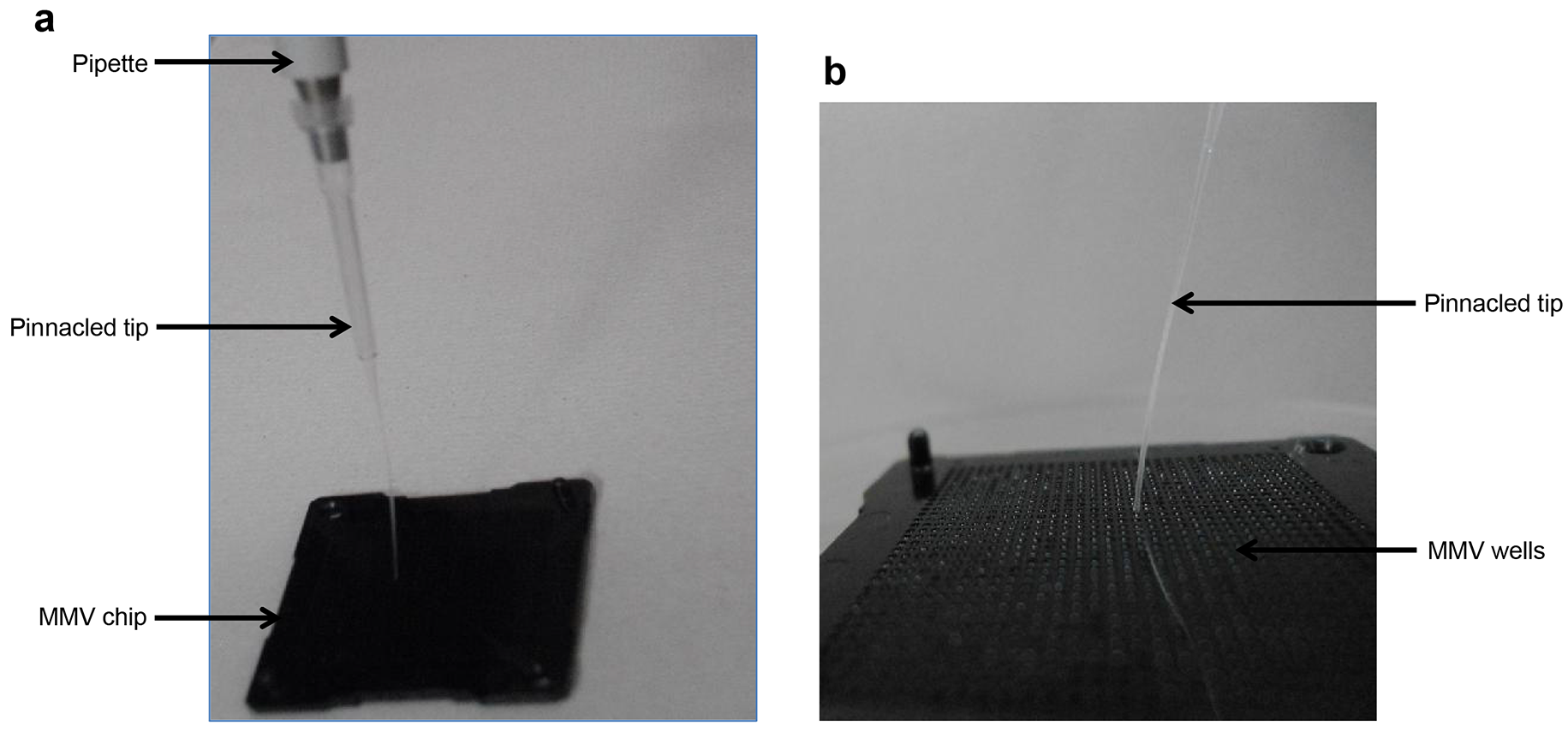

Supplement: Additional file 10: Figure S9 — Pipette-dependent transfer of solution in an MMV well (P-mode operation). (a) Image of a pipette, tip, and MMV chip. (b) Close-up view of manual pipette operation along MMV wells. [file 1472-6750-14-78-S10.tiff]

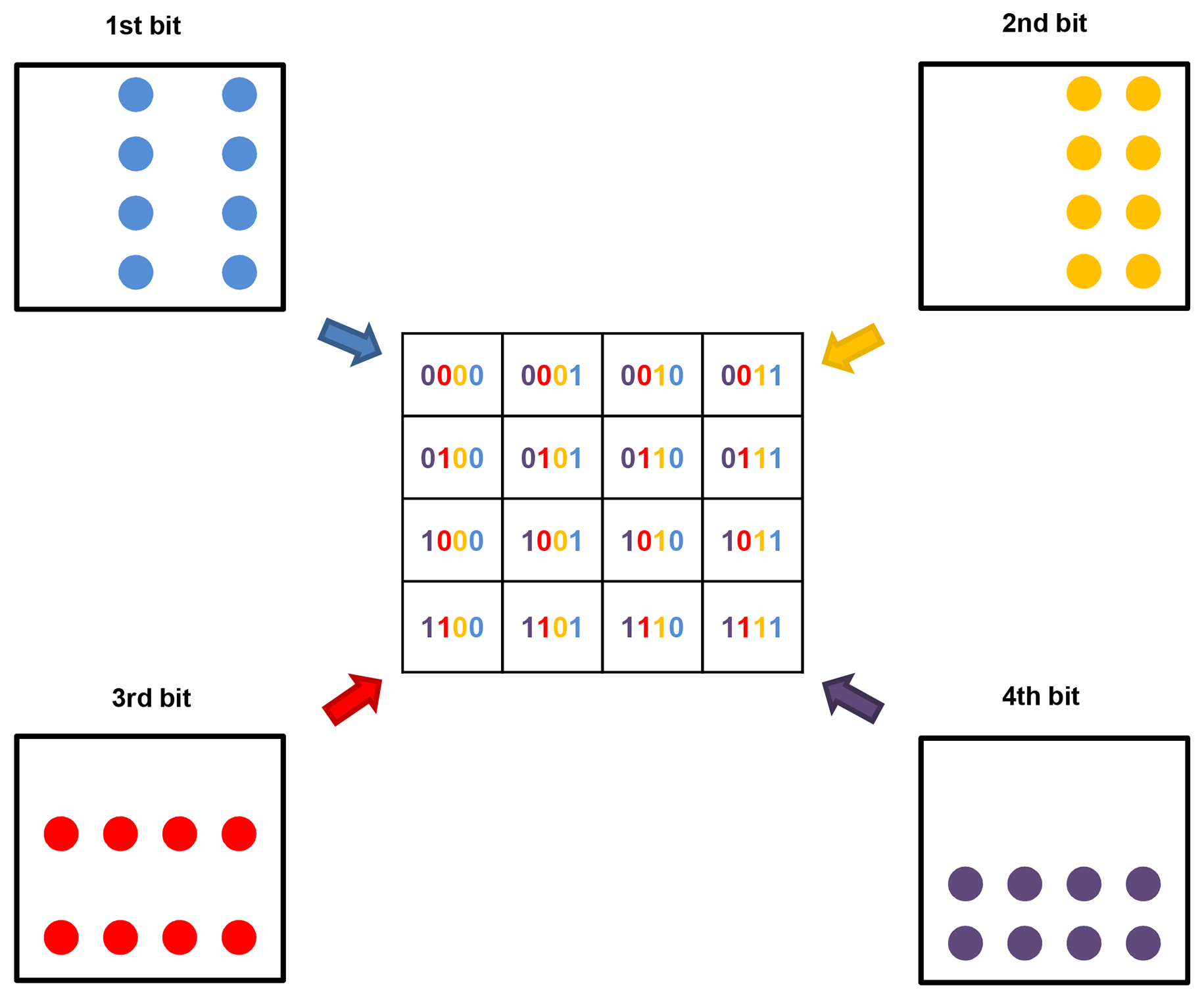

Supplement: Additional file 11: Figure S10 — Generation of diverse conditions. N-times of 2 state (2N) method. Each well of an MMV can be uniquely assigned by a binary number composed of N-bits (here, N = 4). Here, “0” or “1” at each position of a binary number corresponds to the absence or presence, respectively, of a specific component input using the corresponding plate. Thus, “0000” means the absence and “1111” the presence of all four components. Similarly, “0101” implies the presence of only the second and fourth components. [file 1472-6750-14-78-S11.tiff]

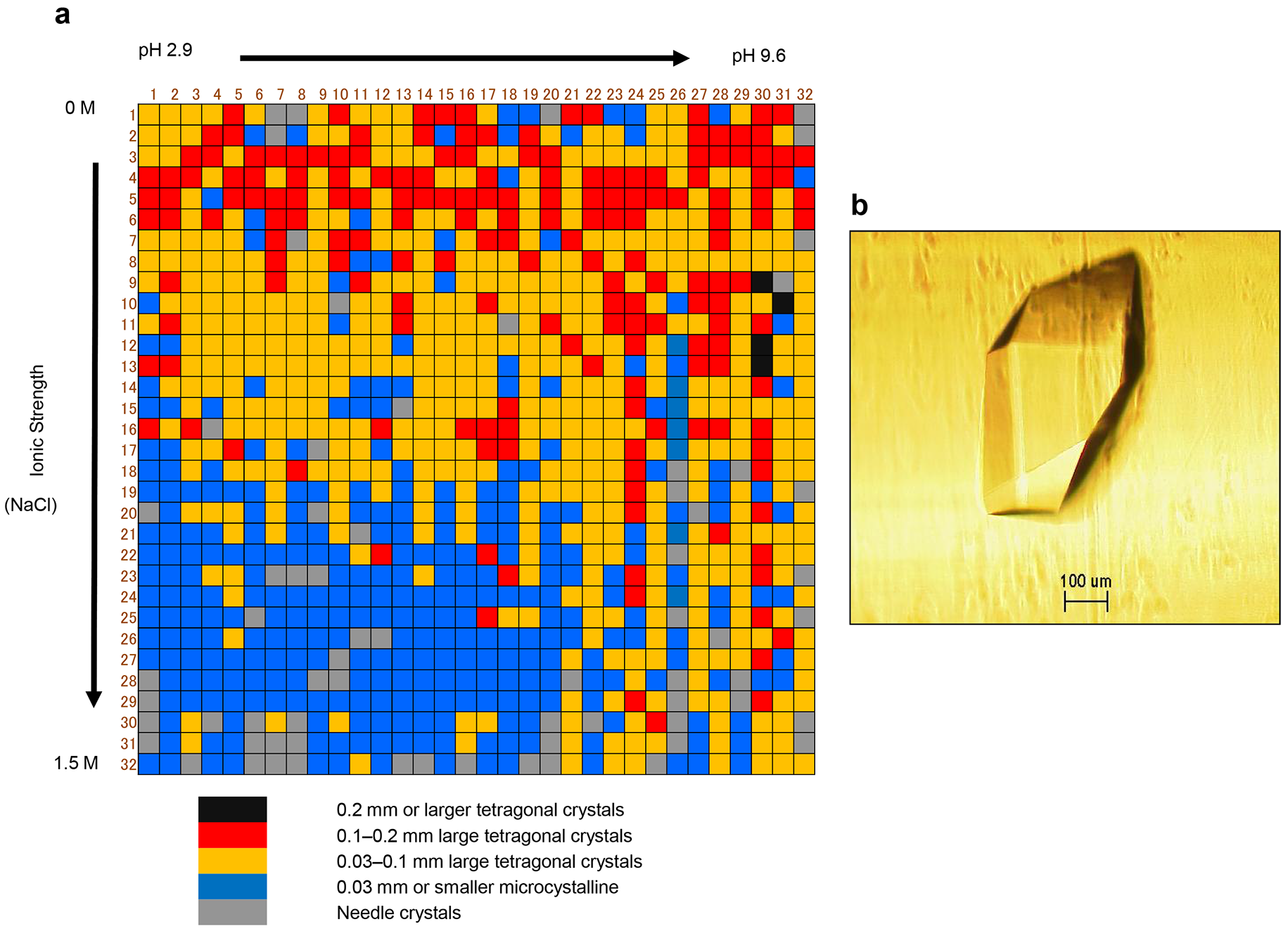

Supplement: Additional file 13: Figure S11 — Phase-diagram-like presentation of lysozyme crystals. (a) Different conditions composed of pH (2.9–9.6) and ionic strength (NaCl: 0–1.5 M) generated different types of lysozyme crystals. Each shape of crystal in MMV wells is depicted in different colors. Four main types of crystals (large and small tetragonal crystals and microcrystalline and needle-like crystals) were observed. (b) Microscopic image of a crystal (0.65 mm in length) obtained by the lysozyme crystallization reproducibility experiment (at 50 μL scale) performed under one of the conditions generated in the MMV chip, i.e., 0.6 M NaCl and pH 8.6. The scale bar is 100 μm. [file 1472-6750-14-78-S13.tiff]

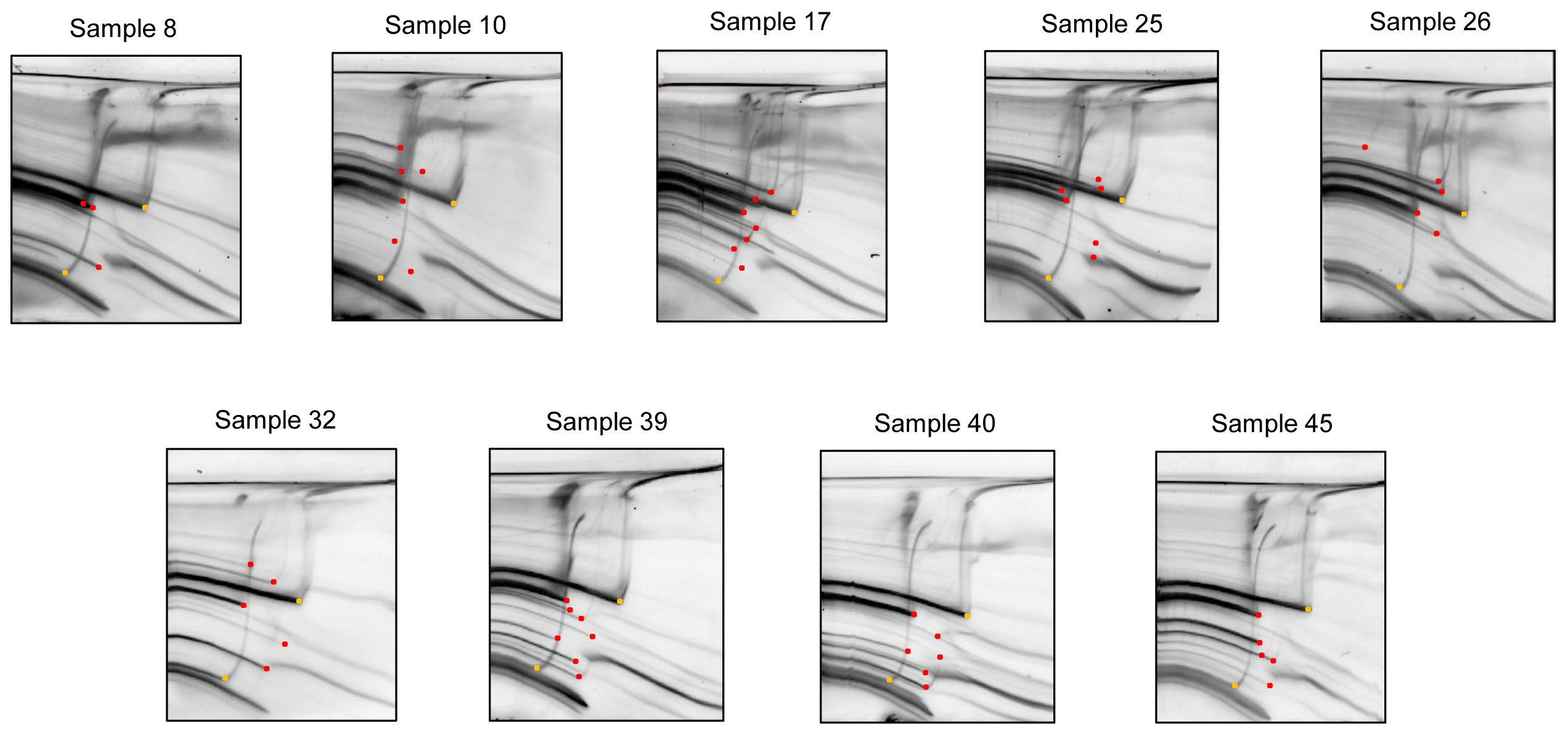

Supplement: Additional file 14: Figure S12 — Original genome profiles obtained from random PCR-successful microbial samples appearing in the MMV-PCR experiment. Samples that have been successfully processed up to clustering analysis (Figure 4d in text) are shown. Sample feature points (pre-spiddos) and internal reference points are indicated by red and yellow dots, respectively. [file 1472-6750-14-78-S14.tiff]

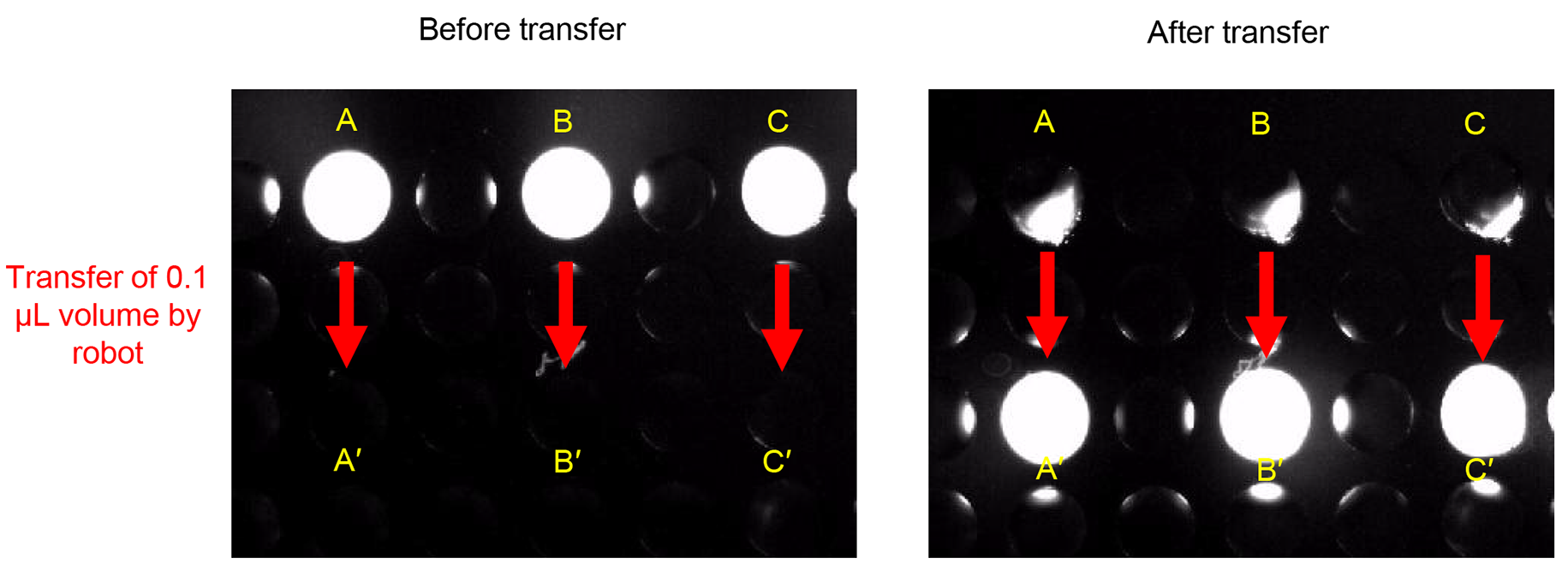

Supplement: Additional file 17: Figure S13 — Well-to-well transfer by nanoliter dispenser robot. Transfer of 0.1 μL volume of solution from upper donor wells (A, B, and C) to lower acceptor wells (A′, B′, and C′) by robotic transfer in the same MMV. In this experiment, around 5% solution (0.005 μL) is left after transfer as shown in the crescent shape (upper row) due to the difficulty of withdrawing all the solution. [file 1472-6750-14-78-S17.tiff]

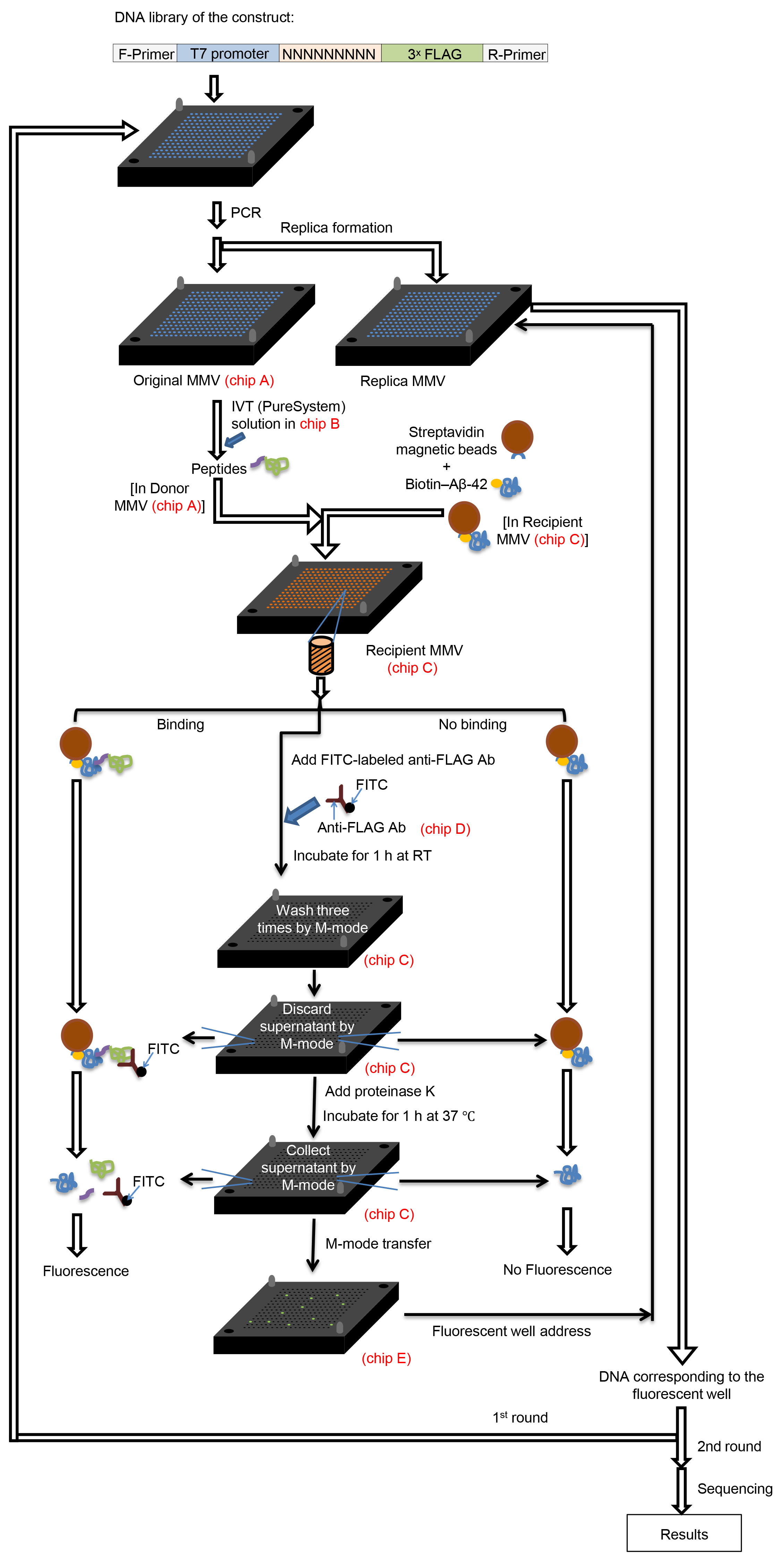

Supplement: Additional file 18: Figure S14 — Micro-high-throughput screening (μ-HTS) of Aβ-binding peptides by panning on microarray MMV (POMM). A DNA library of candidate sequences was transferred to the MMV. Each candidate sequence contained the T7 promoter region and was tagged with 3× FLAG. MMV PCR was performed, and MMV was replicated. The replica MMV was stored (at −20°C) for future replication and screening experiments. For subsequent steps, the original MMV was used. DNA sequences were in vitro transcribed and translated (IVT) (see Additional file 12). Selectively Aβ 42-binding peptides were extracted using streptavidin magnetic beads and biotin-conjugated Aβ 42. MMV was thoroughly washed to eliminate everything except binding peptides. FITC-labeled anti-FLAG antibody solution was added, MMV was washed, and peptides were released by proteinase K treatment. MMV was visualized in the detection unit (CCD camera or TRF unit or laser scanner with FITC filter). In addition, MMV wells stained with FITC indicated the presence of Aβ-42 binding peptides, resulting in fluorescence, whereas in the absence of Aβ-binding peptide, FITC-labeled anti-FLAG antibody did not bind and was washed out in the initial steps, thus resulting in no fluorescence. [file 1472-6750-14-78-S18.tiff]

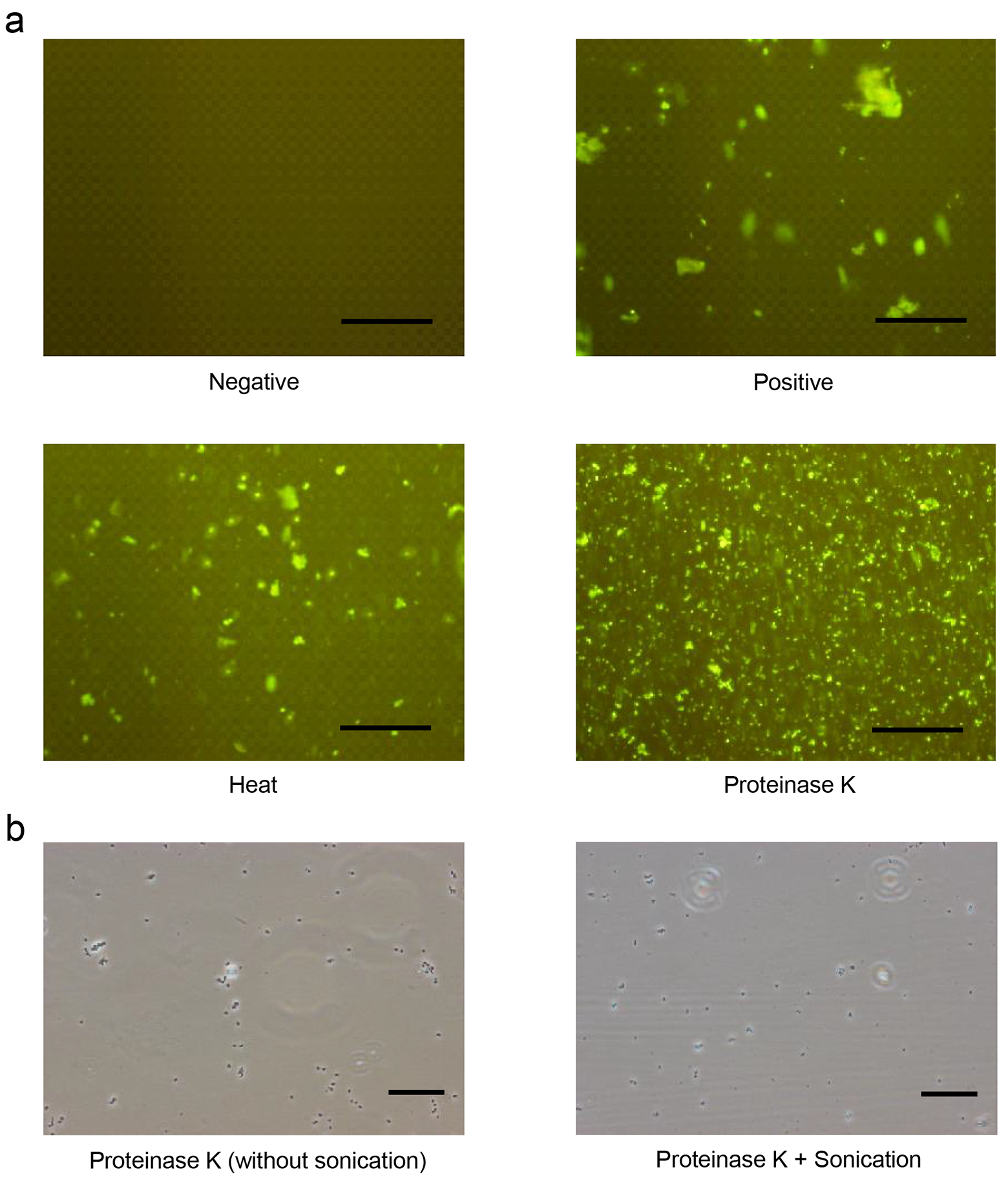

Supplement: Additional file 19: Figure S15 — Deflocculation of an oral microbiome sample. (a) Microscopic view of SYBR gold-stained negative (PBS buffer only), positive (oral microbiome sample without treatment), heat (positive sample with heat treatment only), and proteinase K (positive sample with proteinase K treatment only) samples. (b) Phase-contrast microscopic images of an oral microbiome sample treated with proteinase K only and with both proteinase K and sonication (for 1 min). The scale bar is 50 μm for both (a) and (b). The ratio of single to flocculated cells was approximately 100:1. [file 1472-6750-14-78-S19.tiff]

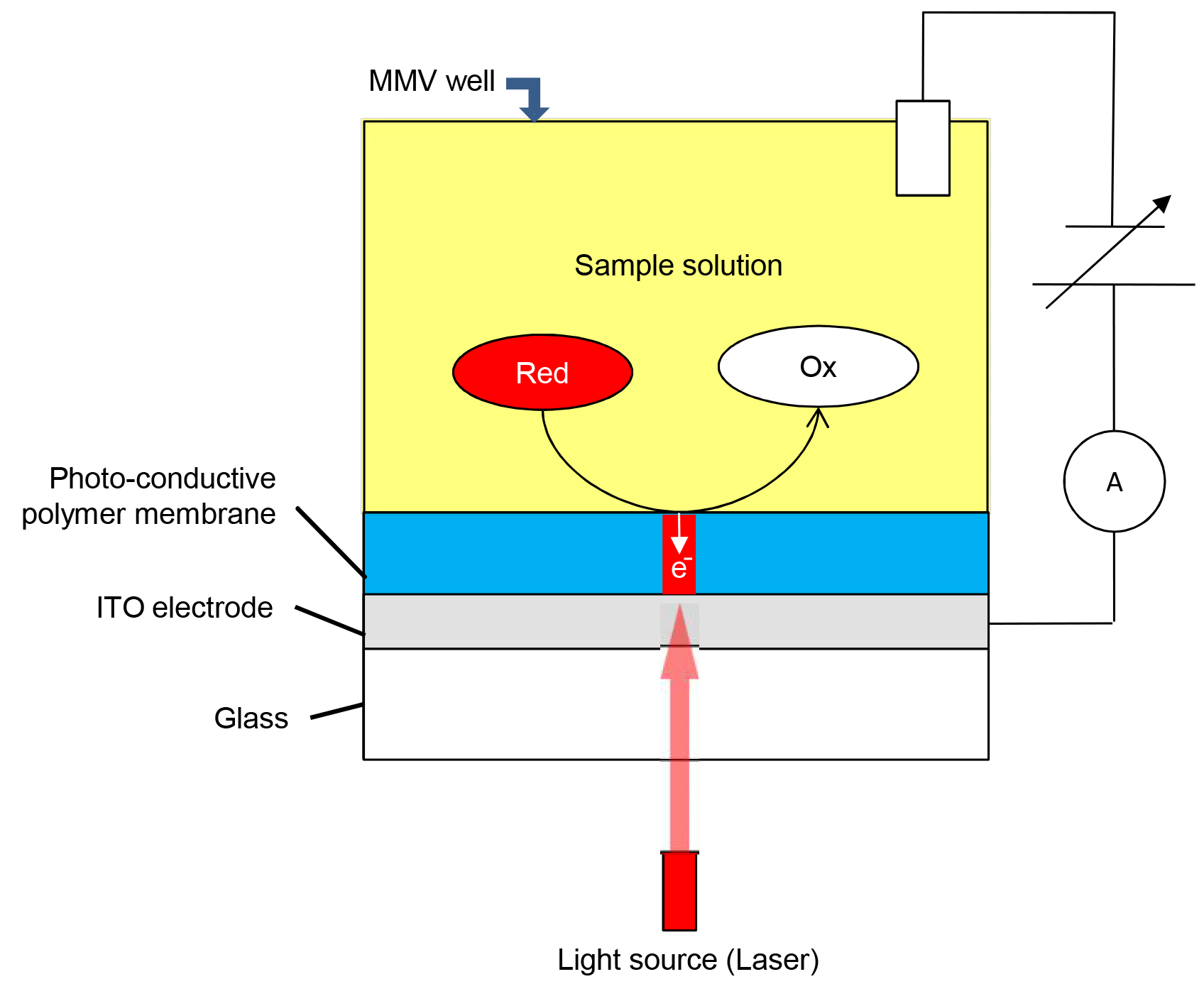

Supplement: Additional file 20: Figure S16 — A trial semiconductor-based apparatus for the evaluation of solution conductivity in MMV wells. The conductivity of the solution in a particular well can be selectively monitored by laser-light illumination through light-transparent semiconductor ITO and photoconductive polymer membrane. The figure shows the redox reaction occurring on the surface of the photoconductive polymer membrane. [file 1472-6750-14-78-S20.tiff]
