# Supplementary material for: Establishment of a reborn MMV-microarray technology: realization of microbiome analysis and other hitherto inaccessible technologies
Source: BMC Biotechnol. 2014 Aug 21;14:78. doi: 10.1186/1472-6750-14-78 (PMC4153446; doi:10.1186/1472-6750-14-78)
Supplement: Additional file 12 — 2N method, genome profiling, μTGGE, sequencing analysis, and peptide aptamer selection: panning on microarray MMV (POMM) methods are described in detail. [file 1472-6750-14-78-S12.docx]

### Additional file 12

**2^N^ method**

The full diversity of a microarray of 2^N^ wells can be created using N different (orthogonal) states (Additional figure 10) [1]. Thus, 2^N^ states can be easily prepared using N’ sheets for packing (N/2 sheets are sufficient if we consider the symmetric relationship among the set of patterns). For instance, when N = 4, two sheets of packing are sufficient and each sheet can be used in two ways (one way and the other that is transverse to this position). As a result, all wells uniquely assigned by the binary number correspond to a mutually different state. For example, the well assigned as 0000 contains none of the components, and the well assigned as 1111 contains all of the components; similarly, the well assigned as 1010 contains the components corresponding to components 1 and 3. Here, we can use a 1024-well microarray (N = 10) for examining 1024 conditions on a sample plate.

**Genome Profiling (GP)**

Genome profiling (GP) comprises three main steps (*i*) random PCR, (*ii*) micro-temperature gradient gel electrophoresis (µTGGE), and (*iii*) computer-aided normalization [2]. Random PCR involves mismatch- or bulge-containing primer–template hybrid structures and leads to random sampling of the whole genomic DNA. In GP, random PCR products are processed using µTGGE analysis, which produces a sequence-specific DNA melting profile for each sample (Figure 4a). The first transition point in each DNA band, denoted as a “feature point,” is processed to generate *spiddos* (species identification dots) with the aid of a computer utilizing internal references. The coordinates of *spiddos* are specific to each genome and correspond to two factors, temperature and mobility, whose values are dependent on both the sequence of the primer and that of the template. In addition, *spiddos* can be used to provide an amount of information sufficient for species identification [3]. The patterns of *spiddos* of two genomes are compared, and it provided a *PaSS* (pattern similarity score) value. A measure of genome distance *d_G_* can be calculated using the following formula:

$$PaSS= 1-\frac{1}{n}\sum_{i=1}^{n} \frac{\left| {P_{i}}^{(1)}- {P_{i}}^{(2)} \right|}{\left| {P_{i}}^{(1)} \right|+\left| {P_{i}}^{(2)} \right|}$$

$$d_{G}=1-PaSS$$

Here, ${P_{i}}^{(1)}$ and ${P_{i}}^{(2)}$ represent the normalized positional vectors (function of temperature and mobility) for *spiddos* ${P_{i}}^{(1)}$ and ${P_{i}}^{(2)}$ from genome (1) and genome (2), respectively, while $i$ designates the serial number of *spiddos*. *PaSS* is 1 for a complete match in two sets of *spiddos*. In general,$0\leq PaSS\leq1$ and thus$0\leq d_{G}\leq1$.

**µTGGE**

Random PCR products were analyzed using µTGGE, a miniaturized version of temperature gradient gel electrophoresis (TGGE) [4]. A slab gel [6% (w/v) of denaturing polyacrylamide gel with 8 M urea] having a size of 1 inch × 1 inch, that is, 2.5 cm × 2.5 cm was used^4^. The PCR product was loaded on the top of the gel along with two internal reference DNAs *i*) internal Ref 1 of 200-bp, (a 191-bp fragment from the bacteriophage fd gene VIII, sites 1350–1540 (approximately) attached to a 9-bp sequence, CTACGTCTC, at the 3′-end; T_m_ of 60 °C under standard conditions) and *ii*) internal Ref 2 of 900 bp taken from pBR322 (T_m_ of 61.4 °C under standard conditions) and subjected to electrophoresis in a µTGGE apparatus (Lifetech) at 100 V for 12 min. A temperature gradient of 15–65 °C perpendicular to the direction of DNA migration was applied. The electrophoresis bands were visualized with SYBR gold staining and imaged with a fluoroimager (Molecular Imager FX, Bio-Rad). Internal reference bands of known melting pattern were used to normalize each genome profile and to obtain *spiddos*.

**Sequencing**

From a genome profile generated by µTGGE, a band of interest from the gel was selected, and a portion of it was picked carefully (avoiding picking nearby bands). Band picking was performed with a vacuum pump and a microtip. This picked band was then reamplified for 30 cycles (denaturation at 94 °C for 30 s, annealing at 60 °C for 60 s, and extension at 74 °C for 60 s) using PCR mix consisting of 320 µM dNTPs (N = G, A, T, or C), 0.7 µM primer pfm 19 (5′CAGGGCGCGTAC3′), 10 mM Tris-HCl (pH 8.3), 50 mM KCl, 1.5 mM MgCl_2_, and 0.025 U/µL Taq DNA polymerase (Takara). The resulting PCR product was ligated to pGEM-T Easy Vector (Promega) at 4 °C overnight. Competent cells of *E. coli* DH5α (purchased from Toyobo) were transformed with the ligation product. Transformed cells were then cultivated on LB agar plates containing 1% tryptone, 0.5% yeast extract, 1% NaCl; (pH 7.0 adjusted with NaOH), 1.5% agar, 10 mg/200 mL ampicillin, 50 mg/mL X-Gal in DMF (20 µL), and 0.1 M IPTG (100 µL). These plates were incubated at 37 °C for 12–14 h. After incubation, white colonies were picked with a sterilized toothpick, transferred into LB broth (1% tryptone, 0.5% yeast extract, and 1% NaCl; pH 7.0), and incubated at 37 °C for 12–14 h with shaking at 180 rpm. After the integration of the gene was confirmed, the plasmid DNA was purified using Wizard™ Plus SV Minipreps DNA Purification System (Promega). The sample thus purified was commercially sequenced (Operon Bio-Technology Co., Ltd.).

**Peptide aptamer selection: panning on microarray MMV (POMM)**

A function-based peptide selection system, termed as panning on microarray MMV (POMM), was performed as follows: peptide-encoding DNAs (both function-known and -unknown in the ratio of 10:10^5^) were charged into MMV wells (100 DNA molecules/well) and PCR-amplified [using a PCR mixture containing 1× Ex Taq buffer, 200 µM dNTPs (N = G, A, T, or C), 0.025 U/µL Ex Taq, and 0.4 µM CA primer] with the MMV chip sealed using 0.76 mm-thick self-adhesive silicone tape for 40 cycles (denaturation at 95 °C for 40 s, annealing at 60 °C for 60 s, extension at 72 °C for 60 s) in a conventional thermocycler (Bio-Rad C1000 Touch).

After PCR, this MMV chip (original, chip-A) was used to generate couple of replica MMVs. The original MMV (chip-A) was processed for the selection (10^2^ different molecules/0.5 µL) experiment, and the other replica MMVs were stored (at −20 °C) for future use. The solution in chip-A was evaporated to nearly half, and IVT (*in vitro* transcription/translation) solution PUREfrex™ composed of solutions I (500 µL), II (50 µL), and III (50 µL) (GeneFrontier Corporation) was added by well-to-well transfer (S-mode) from a volume-controlled MMV (chip-B) (0.25 µL/well). The MMV chip-A was sealed with silicone tape and incubated for 2–4 h at 37 °C.

Additionally, Aβ 42-binding magnetic beads were prepared by incubating a mixture of 700 µL streptavidin magnetic bead suspension (Cosmo Bio, Co., Ltd., Tokyo, Japan) and 150 µL Aβ 42 peptide–biotin conjugate (10 pmol/µL) (Funakoshi Co., Ltd.) in an Eppendorf tube at room temperature for 1 h. This suspension was poured onto a new MMV (chip C) containing PBS buffer and 0.1% Triton-X. After removal of the supernatant from chip C by centrifugation (100 *g*) with magnetic beads attracted to a magnet placed at the bottom of chip C, the IVT product in chip A was transferred to chip C, enabling Aβ 42 and Aβ 42-binding peptides to interact and bind with reciprocal motion of a magnet above and below the MMV chip C for 1 h at room temperature. Separation of beads and solution was performed as described above. By washing three times with the buffer (PBS containing 1 M NaCl and 0.1% Triton-X) with a remnant ratio of 1% per round, all the Aβ 42-unbound peptides were removed, and Aβ 42-binding peptides were recovered on the beads binding to Aβ 42.

A solution containing FITC-labeled anti-FLAG antibody [Monoclonal ANTI-FLAG^®^ M2-FITC (Sigma-Aldrich), 20-fold diluted in PBS containing 0.1% Triton-X], charged into a fresh MMV (chip D), was transferred to chip C. This chip C was sealed, mixed with a magnet, and incubated for 1 h at room temperature to allow binding between FITC-labeled antibody and FLAG-fused peptide. The supernatant was discarded and the remaining beads were washed as described above.

Aβ 42-binding peptides in chip C were released by proteinase K (1/50× concentration, Invitrogen) using PBS containing 0.1% Triton-X and incubated for 1 h at 37 °C. The liquid in chip C was transferred (M-mode) to a fresh MMV (chip E), and the FITC fluorescence of the solution in chip E was detected using a laser scanner with FITC filter.

# References for additional methods

1. Kinoshita Y, Tayama T, Kitamura K, Salimullah M, Uchida H, Suzuki M, Husimi Y, Nishigaki K: **Novel concept microarray enabling PCR and multistep reactions through pipette-free aperture-to-aperture parallel transfer.** *BMC Biotechnol* 2010, **10**:71.
2. Nishigaki K, Naimuddin M, Hamano K: **Genome profiling: a realistic solution for genotype-based identification of species.** *J Biochem* 2000, **128**:107–112.
3. Naimuddin M, Kurazono T, Zhang Y, Watanabe T, Yamaguchi M, Nishigaki K: **Species-identification dots: a potent tool for developing genome microbiology.** *Gene* 2000, **261**:243–250.
4. Biyani M, Nishigaki K: **Hundred-fold productivity of genome analysis by introduction of micro temperature-gradient gel electrophoresis.** *Electrophoresis* 2001, **22**:23–28.
