# Supplementary material for: Establishment of a reborn MMV-microarray technology: realization of microbiome analysis and other hitherto inaccessible technologies
Source: BMC Biotechnol. 2014 Aug 21;14:78. doi: 10.1186/1472-6750-14-78 (PMC4153446; doi:10.1186/1472-6750-14-78)
Supplement: Additional file 15: Table S2 — BLASTN-aided tentative assignment of species for each DNA band in the NNMA experiment. [file 1472-6750-14-78-S15.docx]

**Additional file 15: Table S2.** BLASTN-aided tentative assignment of species for each DNA band in the NNMA experiment.

| **Sample Number^x^** | **Query DNA Size** | **Possible Source Organism**  **(ccgf corresponding gene)** | **Query Coverage (Max. Identity)** | **GenBank Accession Number of the Highest Hit** | **Oral Habitation^y^** |
| --- | --- | --- | --- | --- | --- |
| 8 | 700 bp | *Leuconostoc citreum* KM20 (rplN, rpsQ, and rpmC genes coding for ribosomal proteins L14, S17, and L29, respectively) | 100% (99%) | DQ489736.1 | ○ |
| 10*a* | 700 bp | *Leuconostoc citreum* KM20 (rplN, rpsQ, and rpmC genes coding for ribosomal proteins L14, S17, and L29, respectively) | 100% (99%) | DQ489736.1 | ○ |
| 17 | 712 bp | *Rothia dentocariosa* ATCC 17931 (RNA polymerase sigma factor RpoD and putative ATP-dependent DNA helicase RecG coding genes) | 99% (93%) | CP002280.1 | ◎ |
| 25*a* | 700 bp | *Leuconostoc citreum* KM20 (rplN, rpsQ, and rpmC genes coding for ribosomal proteins L14, S17, and L29, respectively) | 100% (99%) | DQ489736.1 | ○ |
| 32*a* | 700 bp | *Leuconostoc citreum* KM20 (rplN, rpsQ, and rpmC genes coding for ribosomal proteins L14, S17, and L29, respectively) | 100% (99%) | DQ489736.1 | ○ |
| 25*b* | 389 bp | *Phytophthora infestans* T30-4 [conserved hypothetical protein (PITG_09315) coding gene] | 6% (100%) | XM_002903552.1 | ‒ |
| 45 | 384 bp | *Psychrobacter sp*. PRwf-1 (DNA uptake lipoprotein-like protein coding gene) | 46% (71%) | CP000713.1 | △ |
| 10*b* | 430 bp | *Leuconostoc citreum* KM20 (folC2, and folE genes coding for folylpolyglutamate synthase and GTP cyclohydrolase, respectively) | 100% (99%) | DQ489736.1 | ○ |
| 32*b* | 428 bp | *Haemophilus parainfluenzae* T3T1 (L-glutamine:D-fructose-6-phosphate aminotransferase gene) | 100% (93%) | FQ312002.1 | ◎ |
| 26 | 308 bp | *Rothia mucilaginosa* DY-18 DNA (gene coding for ATPase component of various ABC-type transport system) | 100% (95%) | AP011540.1 | ◎ |
| 39 | 299 bp | TPA: *Aspergillus nidulans* FGSC A4 (1,3-beta-D-glucan synthase catalytic subunit putative uncharacterized protein coding gene) | 100% (86%) | BN001302.1 | ○ |
| 40 | 224 bp | *Cronobacter sakazakii* SP291 (GDP-mannose mannosyl hydrolase NudD and glycosyl transferase genes) | 66% (81%) | CP004091.1 | ◎ |

**^x^** Letters *a* and *b* after the sample number indicate two different bands a and b obtained in a single genome profile. The assignment of different species of these DNAs indicates that if the assignment is correct, there were composite genomes in the corresponding well.

**^y^** ◎, Registered as oral organisms in Human Oral Microbiome Database (HOMD); ○, assignable to be oral origin; △, rare but still assignable as oral origin, considering that airborne spores of *Aspergillus nidulans* can be transferred to the mouth cavity by inhalation and cause aspergillosis in the mouth and can infect gums, teeth, and sinuses. *Leuconostoc citreum* is responsible for fermentation of cabbage and found in kimchi. *Psychrobacter* species are considered rare opportunistic human pathogens and have been isolated from specimens obtained from human blood, cerebrospinal fluid, brain tissue, urine, ears, eyes, vulva, wounds, and other cutaneous sources. This gram-negative rod is associated with fish, processed meat, and poultry products. −, Possible contamination or novel origin of sequence, considering that *Phytophthora infestans* is a plant pathogenic fungus, and that hit related to it showed very low query coverage.
