# Supplementary material for: Establishment of a reborn MMV-microarray technology: realization of microbiome analysis and other hitherto inaccessible technologies
Source: BMC Biotechnol. 2014 Aug 21;14:78. doi: 10.1186/1472-6750-14-78 (PMC4153446; doi:10.1186/1472-6750-14-78)
Supplement: Additional file 16: Table S3 — Basic data obtained for three trials of the NNMA experiments. [file 1472-6750-14-78-S16.docx]

**Additional file 16: Table S3.** Basic data obtained for three trials of the NNMA experiments.

| Trial No. | Experiment | Probe Used^a^ | Number of clones tested | Clustering tree generated |
| --- | --- | --- | --- | --- |
| 1 | Preliminary 1 | Hunt | 28 clones for GP and 9 clones for the sequencing test | Strongly consistent as an oral source |
| 2 | Preliminary 2 | Hunt | 10 clones for GP test | Moderately consistent as an oral source |
| 3 | This study | pfm 19 | 47 clones for GP and 9 clones for sequencing test | Strongly consistent as an oral source |

**^a^** Hunt, 5′-TGCTGCTGCTGC-3′; pfM19, 5′-CAGGGCGCGTAC-3′
