# Supplementary material for: Establishment of a reborn MMV-microarray technology: realization of microbiome analysis and other hitherto inaccessible technologies
Source: BMC Biotechnol. 2014 Aug 21;14:78. doi: 10.1186/1472-6750-14-78 (PMC4153446; doi:10.1186/1472-6750-14-78)
Supplement: Additional file 21: Table S4 — Tentative cost comparison of the reagents required in MMV and a 96-well microplate (for 1000 reactions). (a) Cost comparison for a multistep function-based screening experiment. (b) Cost comparison for the experiment of apoptosis detection in HeLa cells. The prices were taken from those in 2012 in Japan for both tables (a) and (b). [file 1472-6750-14-78-S21.docx]

**Additional file 20: Additional Table 4. Tentative cost comparison of the reagents required in MMV and a 96-well microplate (for 1000 reactions).** (a) Cost comparison for a multistep function-based screening experiment. (b) Cost comparison for the experiment of apoptosis detection in HeLa cells. The prices were taken from those in 2012 in Japan for both tables (a) and (b).

**(a)**

|  |  | MMV (0.5 µL/well) | | | | 96-well microplate (50 µL/well) | |
| --- | --- | --- | --- | --- | --- | --- | --- |
| **Name of methods** | **Reagents used** | **Net required amount and price** | | **Working amount and price** | | **Net required amount and price/Working amount and price** | |
|  |  | **Amount (µL)** | **Price (Yen)** | **Amount (µL)** | **Price (Yen)** | **Amount (µL)** | **Price (Yen)** |
| PCR | PCR mixture including SpeedSTAR poly & primers | 500 | 2100 | 800 | 3360 | 50000 | 210000 |
| Transcription/translation | PURESystem | 250 | 14000 | 600 | 33600 | 25000 | 1400000 |
| Binding assay | Biotin labeled Aβ-42 | 0.1 mM | 500 | 0.2 mM | 1000 | 0.1 mM | 50000 |
|  | Magnotex-SA bead | 100 | 3100 | 250 | 7750 | 10000 | 310000 |
|  |  | **Total:** | 19700 |  | **45710** |  | **1970000** |

**(b)**

|  |  | MMV (0.5 µL/well) | | | | 96-well microplate (50 µL/well) | |
| --- | --- | --- | --- | --- | --- | --- | --- |
| **Name of methods** | **Reagents used** | **Net required amount and price** | | **Working amount and price** | | **Net required amount and price/Working amount and price** | |
|  |  | **Amount (µL)** | **Price (Yen)** | **Amount (µL)** | **Price (Yen)** | **Amount (µL)** | **Price (Yen)** |
| Cell culture | D-MEM including FBS & antibiotics (for HeLa cells) | 500 | 5 | 800 | 8 | 50000 | 500 |
| Fluorescence stain | Apo-ONE™ Homogenous Caspase-3/7 | 250 | 1500 | 600 | 3600 | 25000 | 150000 |
|  |  | **Total:** | 1505 |  | **3608** |  | **150500** |
